# Impact of Nutrient Imbalance on Wine Alcoholic Fermentations: Nitrogen Excess Enhances Yeast Cell Death in Lipid-Limited Must

**DOI:** 10.1371/journal.pone.0061645

**Published:** 2013-04-26

**Authors:** Catherine Tesnière, Pierre Delobel, Martine Pradal, Bruno Blondin

**Affiliations:** 1 INRA, UMR1083, Montpellier, France; 2 SupAgro, UMR1083, Montpellier, France; 3 Université Montpellier 1, UMR1083, Montpellier, France; Fundação Oswaldo Cruz, Brazil; Maya-Monteiro

## Abstract

We evaluated the consequences of nutritional imbalances, particularly lipid/nitrogen imbalances, on wine yeast survival during alcoholic fermentation. We report that lipid limitation (ergosterol limitation in our model) led to a rapid loss of viability during the stationary phase of fermentation and that the cell death rate is strongly modulated by nitrogen availability and nature. Yeast survival was reduced in the presence of excess nitrogen in lipid-limited fermentations. The rapidly dying yeast cells in fermentations in high nitrogen and lipid-limited conditions displayed a lower storage of the carbohydrates trehalose and glycogen than observed in nitrogen-limited cells. We studied the cell stress response using *HSP12* promoter-driven *GFP* expression as a marker, and found that lipid limitation triggered a weaker stress response than nitrogen limitation. We used a *SCH9*-deleted strain to assess the involvement of nitrogen signalling pathways in the triggering of cell death. Deletion of *SCH9* increased yeast viability in the presence of excess nitrogen, indicating that a signalling pathway acting through Sch9p is involved in this nitrogen-triggered cell death. We also show that various nitrogen sources, but not histidine or proline, provoked cell death. Our various findings indicate that lipid limitation does not elicit a transcriptional programme that leads to a stress response protecting yeast cells and that nitrogen excess triggers cell death by modulating this stress response, but not through *HSP12*. These results reveal a possibly negative role of nitrogen in fermentation, with reported effects referring to ergosterol limitation conditions. These effects should be taken into account in the management of alcoholic fermentations.

## Introduction

The nutrients available for yeasts in grape musts have a major impact on the kinetics of alcoholic fermentations. Yeast is more or less active (with variable fermentation rates) and is able or not able to withstand the stress of alcoholic fermentation (high ethanol concentrations, low pH), according to the availability of nutrients. Nitrogen is a particularly significant nutrient [1], [2], because its availability determines the fermentation rate and to a large extent, the fermentation duration [3]. Lipids are also key nutrients in alcoholic fermentation and oxygen is required for the synthesis of sterols and unsaturated fatty acids [4], [5]. Limitations of unsaturated fatty acids or sterols have negative effects on the maintenance of viability at the end of fermentations [6], [7]. Indeed, some wine making practices, such as strong clarification of musts, can lead to such compounds becoming limiting and are associated with a loss of yeast cell viability.

Little is known about the molecular mechanisms involved in yeast cell death under such conditions and yeast cell death has been only described in terms of the consequences of unsuitable membrane composition. Indeed, the plasma membrane is considered as the major target of ethanol toxicity, and both sterols and unsaturated fatty acids act as regulators of membrane function thereby protecting against the deleterious effects of ethanol [8], [9]. On another side, recent studies have reported that the ability of yeasts to survive starvation depends on how they enter into the state of starvation and on the nature of the limiting nutrient [10]. The adaptation to nutritional deficiencies is affected by the activity of various signalling pathways, including TOR [11], [12] or PKA [13], and pathways that initiate sensing of nutrients directly at the plasma membrane level [14]. In a nutrient-rich environment, the nutrient-sensing pathways TOR/Sch9p and RAS/PKA promote growth and repress the stress response and autophagy. At low ammonium or amino acid concentrations, cell survival, or chronological lifespan (CLS), is extended [15], [16]. It is unclear whether the activity of these signalling pathways affects cell behaviour in situations of nutrient disequilibrium in alcoholic fermentation.

Based on recent theoretical analyses of yeast starvation biology, we explored the molecular mechanisms and the signalling pathways involved in triggering cell-death in response to disequilibrium of lipid/nitrogen nutrients in alcoholic wine fermentation. We show that cell death in lipid-limited fermentations is strongly modulated by the availability of nitrogen. Molecular analyses indicated that TOR nitrogen cellular signalling is involved in triggering cell death in such conditions. Our results demonstrate that the nitrogen status of grape musts is a strong determinant of the outcome of alcoholic fermentations in conditions of lipid limitation.

## Results

### The yeast cell viability is modulated by nitrogen/lipid imbalances

**Figure 1:**
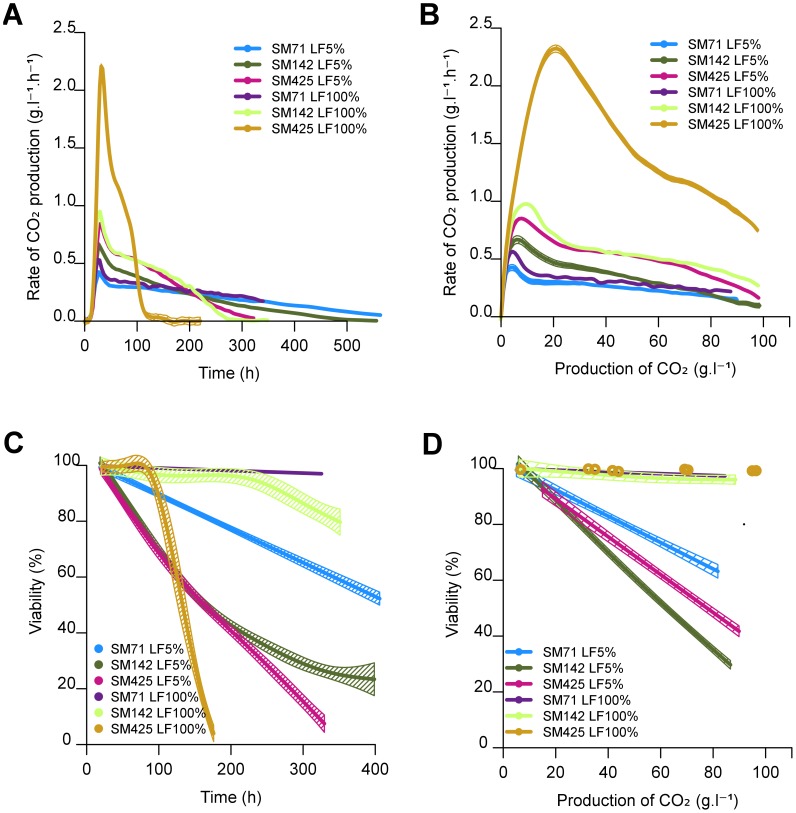
Effect of nutrient imbalances during alcoholic fermentation. Rate of CO_2_ production by *S. cerevisiae* EC1118 cultures at 24°C (A) at various times of fermentation or (B) CO_2_ production, and viability of *S. cerevisiae* EC1118 cells according to (C) time of alcoholic fermentation at 24°C and (D) CO_2_ production. The synthetic medium contained 71 mg/L (SM71) or 142 mg/L (SM142) assimilable nitrogen, and 5% or 100% lipid factors (LF5% or LF100%). Viability was measured by flow cytometry after propidium iodide staining. The graphs are the result of smoothing of measurements series (at least 3 repetitions) using the software R. Smoothing obtained is framed by a confidence interval calculated at 95%.

We examined the effects of the nitrogen and lipid status on the behaviour of the wine yeast EC1118 in alcoholic fermentation. The EC1118 strain was fermented in synthetic medium SM425, SM142 or SM71 (containing 425, 142 and 71 mg/L of assimilable nitrogen, respectively) with either high (LF100%) or low (LF 5%) levels of lipid factors (LF). As expected, when lipids were not limiting, the amount of nitrogen had a significant effect on the rate of fermentation, with a maximal peak of CO_2_ production at 2.25, 1 and 0.59 g/L/h, respectively, and a fermentation period lasting 110 to 340 h (Figure 1A and B). In lipid-limited medium (LF5%), the Vmax of the yeast fermentation was lower, from 0.8 to 0.4 g/L/h, depending on the nitrogen content.

The cell population densities (Table 1) were between 39 and 49 10^6^ cells/mL in lipid-limited conditions (LF5%), the lipid availability being the growth limiting factor (Figure S1). By contrast, the cell population density was far higher in SM425, SM142 and SM71 with LF100% (from 202 10^6^ cells/mL to 68 10^6^ cells/mL). A two-way ANOVA on the log population shows a high significant effect of lipid and of nitrogen, with a significant interaction effect (P-value < 0.001). Moreover, the Wilcoxon rank-sum test shows significant differences (P-value < 5%) for each condition taken two by two.

**Table 1:** Cell population densities determined by flow cytometry at stationary phase.

Strain	Synthetic medium composition		Cell population (10^6^ cell/mL)
	Assimilable nitrogen (mg/L)	Lipid factor (%)	
EC1118	425	100	202.6 ± 9.9
EC1118	425	5	49.2 ± 4.5
EC1118	142	100	106.5 ± 12.3
EC1118	142	5	44.6. ± 6.3
EC1118	71	100	68.4 ± 4.6
EC1118	71	5	39.5 ± 8.8

Cell viability (Figure 1C and D) was high (more than 98%) during the stationary phase in media rich in lipids (LF100%) irrespective of the initial nitrogen content (SM425, SM142 or SM71). However, yeast viability was lower in all the fermentations in lipid-limited medium (LF5%). As the fermentation rates were highly variable (Figure 1A), we compared cell viabilities according to the progress of the fermentation, i.e. the production of CO_2_ (Figure 1D), rather than to time.

In lipid-limited fermentations, cell viability decreased regularly with a constant slope for each condition (Figure 1D). This behaviour is consistent with an ethanol-related loss of viability and not chronological aging-related loss of viability. In fact in our conditions, time is not relevant, but ethanol increase (directly proportional to the amount of CO_2_ released during fermentation) is, likely because it is a well-known stressor. Therefore, these findings could indicate that cell death was a direct consequence of ethanol toxicity, although it is not excluded that other factors present in the medium could also be involved. The decrease in cell viability varied depending on the nitrogen content of the medium (Figure 1C and D). Viability remained high in SM71 (70% at 70 g/L of CO_2_ produced), but decreased to a lower level in SM142 (45% at 70 g/L of CO_2_ produced), demonstrating a negative effect of nitrogen availability on the cell viability in lipid-limited fermentations. High nitrogen effect on cell death is strongly dependent on the lipid content and it triggers cell death only when the lipid content is low. Surprisingly the highest nitrogen concentration (425 mg/L) led to an intermediate rate of cell death. Thus, the effect was not strictly dependent on the nitrogen concentration suggesting “complex effect” of nitrogen sources.

The nitrogen sources used in this study were a mixture of amino acids and ammonium, and mimicked a natural must. The nitrogen sources were monitored during the fermentations. All amino acids, except proline, were up taken from SM71 and SM142 at 20 g of CO_2_ (Table S1). As the populations were similar in all fermentations, these nitrogen sources were not used to make new cells. Note that, cell death differed between these cultures, with the lipid content supposed to be the same, excluding the involvement of lipids in the differences. However, in cultures in SM425, most of the amino acids were not totally metabolised (Table S1). This suggests that some residual nitrogen sources may trigger a protective effect in these conditions.

**Figure 2:**
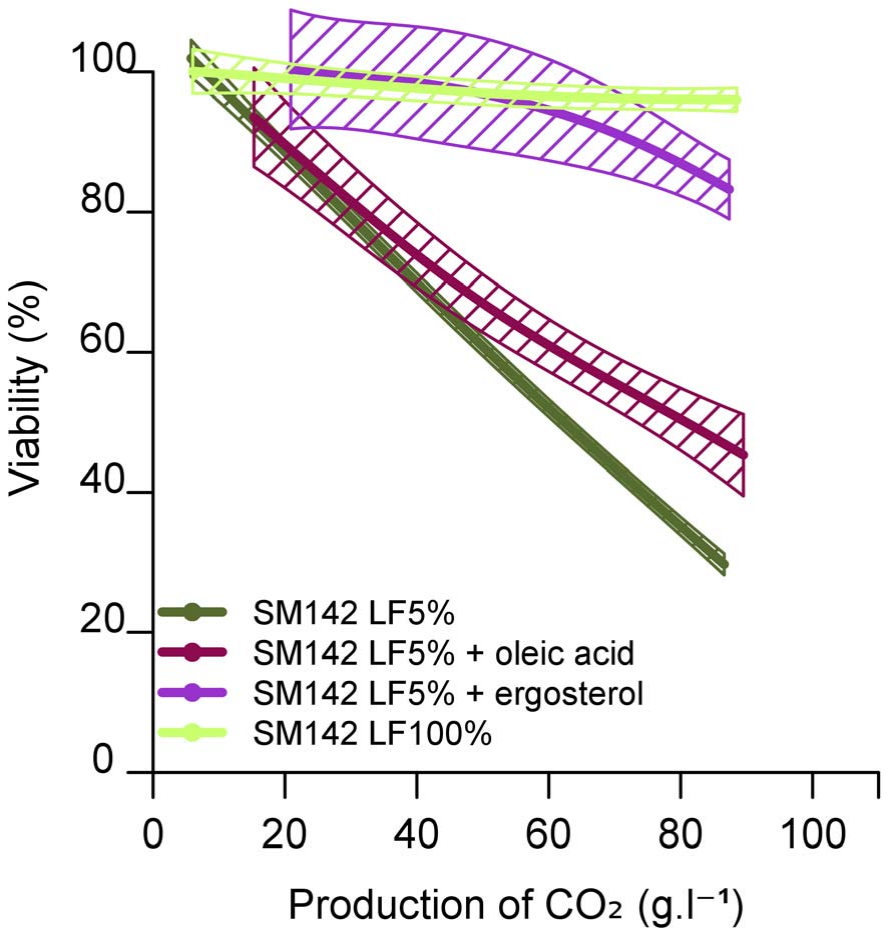
Effect of lipid factors on the viability of *S. cerevisiae* EC1118 cells during alcoholic fermentation. The synthetic medium contained 142 mg/L assimilable nitrogen (SM142) and 5% lipid factor (LF5%), with or without additional oleic acid or ergosterol (content as in LF100%). Viability was measured by flow cytometry after propidium iodide staining. The graphs are the result of smoothing of measurements series (at least 3 repetitions) using the software R. Smoothing obtained is framed by a confidence interval calculated at 95%.

The source of LF used contained a mixture of ergosterol and oleic acid, and we investigated which of these lipids was the limiting factor for growth. Fermentation variables were measured in SM142 LF5% supplemented with oleic acid or ergosterol so that the final lipid concentration was similar to a non-limited medium (LF100%). The addition of oleic acid had only a small effect on the final population of cells, whereas the addition of ergosterol led to an increase in the population (77 10^6^ cells/mL in SM142 LF5% plus ergosterol) (Table 2). The addition of ergosterol also increased cell viability (Figure 2) to close to 100%, and rate of CO_2_ production (Figure S2). Ergosterol was therefore the lipid limiting growth under our experimental conditions.

**Table 2:** Effect of treatment on cell population densities as determined by flow cytometry at stationary phase.

Strain	Synthetic medium composition		Comparison low	Cell population (10^6^ cell/mL)	Comparison high
	Assimilable nitrogen (mg/L)	Lipid factor (%)			
EC1118	142	100	**	106.5±12.3	high
EC1118	142	5	low	44.6±6.3	**
EC1118 hsp12::GFP	142	5	NS	57.5±6.7	NS
EC1118	142	5 + oleic acid	*	36.6±0.6	NS
EC1118	142	5 + ergosterol	*	77.3±6.7	NS
EC1118	142 + rapamycin	5	NS	46.6±2.7	NS
EC1118	142	5	*	44.6±6.3	high
EC1118	71	5	low	39.5±8.8	*
EC1118 hsp12::GFP	71	5	*	48±2.2	NS
EC1118	142 (71 mix + 71	5	NS	47.6	NS
EC1118	NH_4_^+^)	5	NS	42.1±1	NS
EC1118	142 (71 mix + 71 Arg)	5	NS	48.4±1.8	NS
EC1118	142 (71 mix + 71 Glu)	5	NS	44.3±4.1	NS
EC1118	142 (71 mix + 71 Gln)	5	NS	37.1±2.5	*
EC1118	142 (71 mix + 71 His)	100	NS	53.3	NS
EC1118	142 (71 mix + 71 Pro)		*	68.4±4.6	*
	71				

The log population of each condition is compared to the two standards conditions (indicated as “low” and “high”) with the Wilcoxon rank-sum test (*: P-value <5%; **: P-value < 1%).

### The viability of wine yeasts under lipid limitation depends on the source of nitrogen

**Figure 3:**
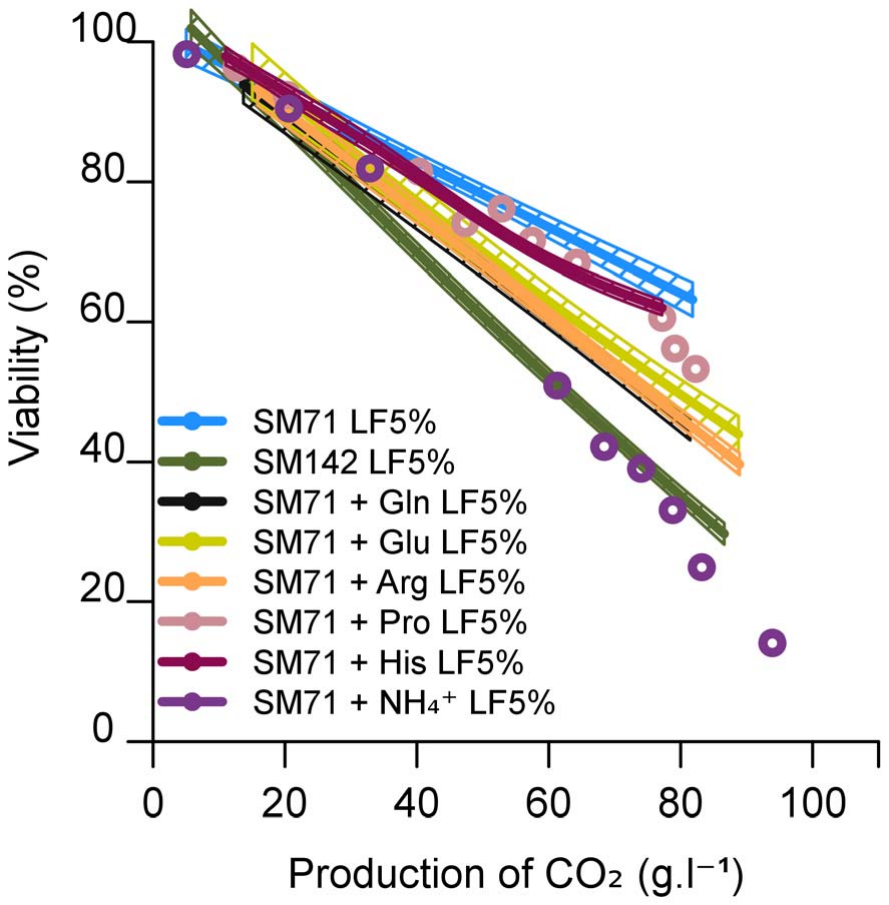
Effect of nitrogen source on the viability of *S. cerevisiae* EC1118 cells during alcoholic fermentation. The synthetic medium contained 71 mg/L assimilable nitrogen (SM71) and 5% lipid factors (LF5%), with or without additional arginine, glutamine, glutamate, histidine or proline (142 mg/L assimilable nitrogen final content as in SM142). Viability was measured by flow cytometry after propidium iodide staining. The graphs are the result of smoothing of measurements series (at least 3 repetitions) using the software R. Smoothing obtained is framed by a confidence interval calculated at 95%.

The combination of lipid limitation and nitrogen excess affected yeast cell viability. We therefore investigated the capacity of amino acids and ammonium to trigger cell death. We considered the amino acids most likely to be available on the basis of their natural abundance in musts and the ability of yeast to use them quickly (arginine, glutamine, glutamate, histidine or proline). SM71 was independently supplemented with each of various amino acids or ammonium, such that the final concentration of assimilable nitrogen was the same as that in SM142. Supplementation with histidine or proline had little or no effect on the rate of fermentation, consistent with the absence or only limited use of these amino acids. The other nitrogen sources triggered a significant increase in the rate of fermentation (Figure S3). Only small variations in final cell populations were observed (from 37 to 53 10^6^ cells/mL) consistent with a tight control of growth by limiting ergosterol (Table 2). However, the addition of the nitrogen sources arginine, glutamine, glutamate or ammonium increased mortality (Figure 3). Thus, an excess of various nitrogen sources enhanced cell death under conditions of lipid limitation. The differences observed seem to be related to the ability of yeast cells to use the supplementary nitrogen source: ammonium was the most effective trigger of cell death with less than 20% viability at the end of fermentation. This effect is consistent with the observation that NH_4_^+^ is toxic for cells deprived for auxotrophy-complementing amino acids, although it is also toxic for cells grown in excess of amino acids. In addition, NH4^+^ reduced the yeast chronological life span by activating of the TOR/PKA pathways and by inhibiting Sch9 [17]. In fact, NH_4_^+^ like other nitrogen sources metabolized, triggers cell death in relation with its signaling capacity on TOR pathway. This leads to a reduction of the stress response and yeast protection against ethanol. Thus, there could be a combination effect of ethanol and NH4^+^, with ethanol acting as a permanent stressor but not being the initial trigger of cell death. However, several different sources of nitrogen increased cell death in our conditions, suggesting that the underlying mechanisms may be different.

### Stress response-dependent sugar storage is reduced in lipid-limited cells

**Figure 4:**
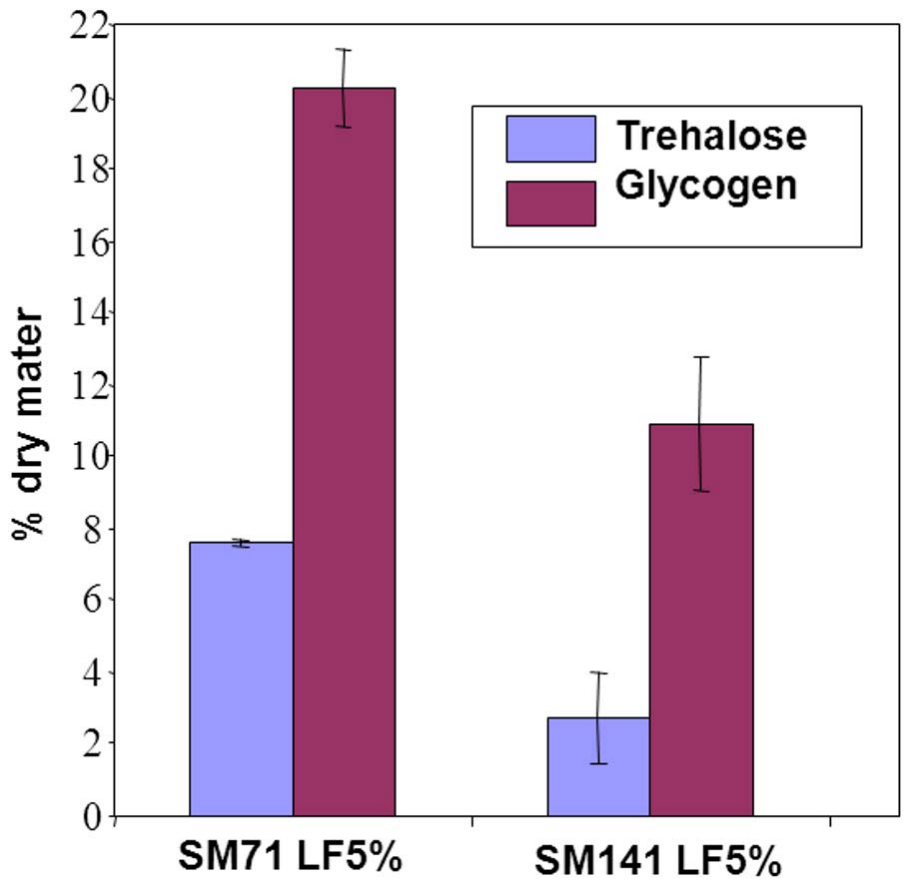
Trehalose and glycogen contents at stationary phase of *S. cerevisiae* EC1118. Cells were fermented in synthetic medium containing 71 mg/L (SM71) or 142 mg/L (SM142) assimilable nitrogen, and 5% or 100% lipid factors (LF5% or LF100%).

Trehalose and glycogen accumulate in response to stress and can be used as indicators of the stress response in yeast. We therefore analysed the content of trehalose and glycogen in yeast stationary phase as markers of the stress response to nutrient limitations. Both compounds were more abundant in cells fermenting in SM71 LF5% than in SM142 LF5% (Figure 4). These results are consistent with a control of the stress response by nitrogen level and suggest that growth limitation by ergosterol does not trigger similar stress response.

### Nitrogen signalling is involved in triggering cell death in response to nitrogen/lipid imbalances

**Figure 5:**
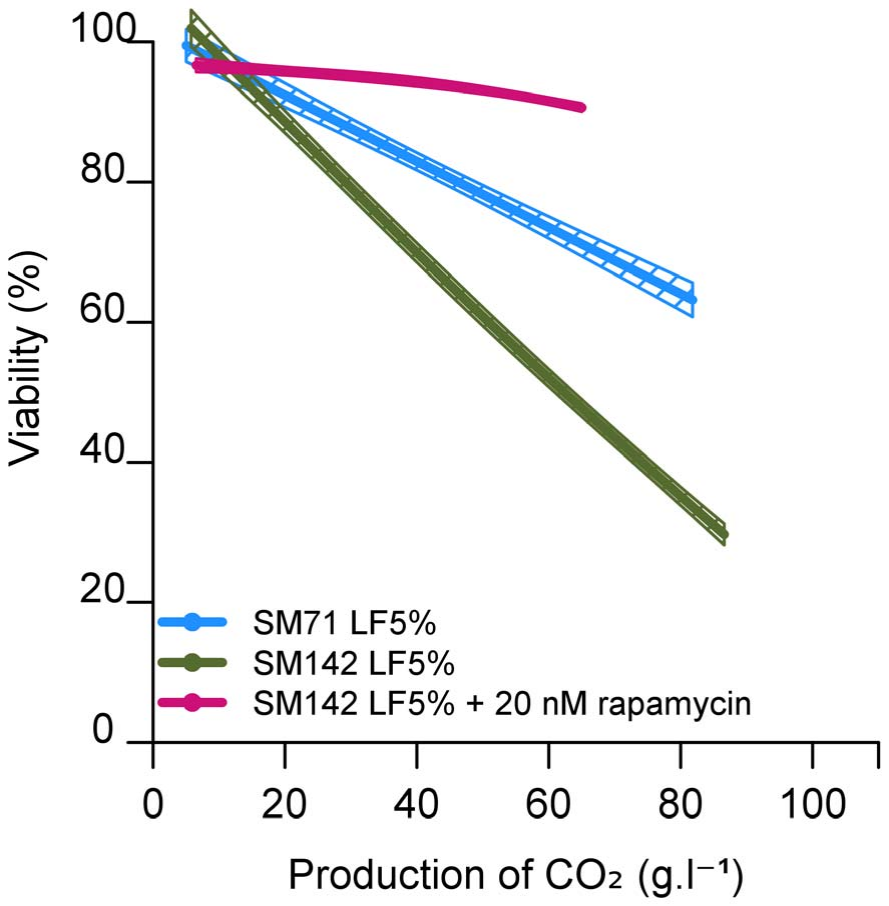
Effect of rapamycin on the viability of *S. cerevisiae* EC1118 cells during alcoholic fermentation. ****** **** **** The synthetic medium contains 142 mg/L assimilable nitrogen (SM142) and 5% lipid factors (LF5%), with or without 20 nmol/L rapamycin. Viability was measured by flow cytometry after propidium iodide staining. The graphs are the result of smoothing of measurements series (at least 3 repetitions) using the software R. Smoothing obtained is framed by a confidence interval calculated at 95%.

**Figure 6:**
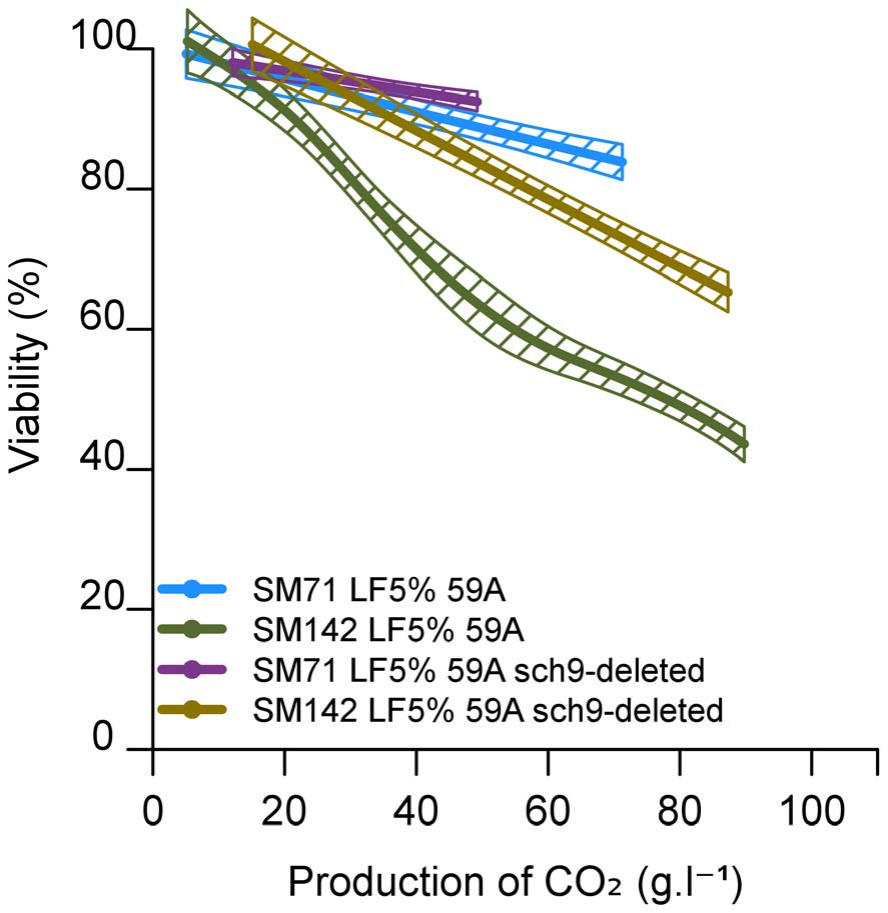
Effect of Sch9 deletion on the cell viability of haploid *S. cerevisiae* 59A during alcoholic fermentation. The synthetic medium contains 71 mg/L (SM71) or 142 mg/L (SM142) of assimilable nitrogen, and 5% lipid factor (LF5%). Viability was measured by flow cytometry after propidium iodide staining. The graphs are the result of smoothing of measurements series (at least 3 repetitions) using the software R. Smoothing obtained is framed by a confidence interval calculated at 95%.

The TOR signalling pathway responds to nitrogen sources and controls various processes that affect the stress resistance and longevity of yeast cells. The TOR kinase, TORC1 (Target Of Rapamycin Complex 1), part of the TOR signalling pathway, is specifically inhibited by rapamycin [18]. The inclusion of 20 nmol/L rapamycin in cultures from inoculation of SM142 LF5% significantly increased the cell viability, to nearly 100 % (Figure 5). This implicates TOR signalling pathway in the cellular response leading to cell death. We then investigated whether *SCH9* was involved. Sch9p is a protein kinase that plays a key role in controlling cell survival and integrates signals from TOR, PKA and other regulators [19], [20]. We assessed the consequences of deletion of the *SCH9* gene from a haploid derivative of EC1118, 59A (strain *sch9::KanMX* 59A). We examined the response of the *SCH9*-deleted strain to nutritional imbalances and its effect on cell viability. In SM71 LF5% the production rate of CO_2_ by the *SCH9*-deleted strain was similar to that of the non-deleted controls (data not shown), and viability was similar. However, in SM142 LF5% the viability of the *SCH9*-deleted strain was significantly greater than that of the wild-type strain, reaching 80% versus 60% in the wild type strain at 60 g of CO_2_ produced (Figure 6). Thus, deletion of *SCH9*, like inhibition of TOR by rapamycin, favoured survival in conditions of lipid/nitrogen imbalance. This suggests that cell death in such nutritional conditions is controlled by Sch9p and TOR activity. Sch9p regulates the expression of a set of genes [21]–[23]. To identify target genes that may contribute to protect against cell death under our conditions, we evaluated the transcriptome changes associated with *SCH9* deletion.

### Global expression changes in the SCH9 mutant under lipid limitation

We performed a genome-wide analysis of the effect of the sch9-deletion on gene expression using DNA microarrays. We compared the expression profiles of wild-type cells (59A) and the sch9-deleted mutant during fermentation at 35 g CO_2_: 104 genes were found to be down-regulated (P-value < 0.05; log FC <−1) and 134 genes up-regulated (P-value < 0.05; log FC >1) in the sch9 mutant (Table S2 and Table S3). Most of the non-redundant GO terms that were significantly associated with the down-regulated gene list were related to ergosterol, sterol, steroid, lipid, isoprenoid and, isopentyl diphosphate biosynthetic processes (Table 3). Almost all the genes encoding proteins involved in ergosterol biosynthesis (*ERG1, ERG2, ERG3, ERG5, ERG6, ERG8, ERG10, ERG11, ERG12, ERG13, ERG20, HMG1*) and other lipid-related genes (12 genes), were down-regulated. Thus, sch9-deletion significantly affected lipid metabolism. Other down-regulated genes were ribosome biogenesis and rRNA processing genes (19 genes), nucleotide-related genes (6 genes), nitrogen-related genes (8 genes) and genes encoding permeases (9 genes). These findings confirm the interrelationship between the TORC1 signaling pathway and the lipid metabolism. They are consistent with the regulation of ribosome biogenesis and RNA processing by Sch9 [23], [24], but in contrast to the genes involved in the ergosterol biosynthesis pathway in non-lipid limited conditions [25]. Significant up-regulation of genes coding for proteins involved in the response to stress (*SSA3, TIP1, HSP30, FMP45, SSA4, HSP12, CTT1, SKN7, XBP1, HSP104, DDR2, GRE1, HAL1*), oxidoreductase activity (18 genes), cell adhesion molecule binding (11 genes), the MAPK signalling pathway and mating (8 genes) were observed in sch9-deleted mutant (Table 4). Therefore, deletion of SCH9 increased the stress response in lipid-limited conditions. Interestingly, most of the up-regulated genes encoded cell wall or plasma membrane proteins, *HXT5, AGA1, SCW10* and *HSP12* being among the most strongly over-expressed genes. It is also interesting to note that, related to the increase of *HXT5* expression observed in the present study, this hexose transporter has also been described, among the most physiological-relevant transporters, as being the less inhibited by ethanol [26].

**Table 3:** GO term annotations for genes significantly down-regulated in the 59A sch9-deleted mutant.

GO category	GO no.	P*-*value	GO description	k	f
Biological process	GO:0006696	<1e-14	ergosterol biosynthetic process	12	23
	GO:0016126 GO:0006694 GO:0008610 GO:0008299 GO:0019287	<1e-14	sterol biosynthetic process	14	29
	GO:0008152	<1e-14	steroid biosynthetic process	12	25
		6.403e-13	lipid biosynthetic process	13	52
		1.457e-10	isoprenoid biosynthetic process	7	12
		3.797e-06	isopentenyl diphosphate biosynthetic process	3	3
		7.835e-06	metabolic process	20	425
Cellular component	GO:0005730	3.722e-05	nucleolus	14	253

k: number of genes in the family identified as affected in the experiment; f: total number of genes in the family.

**Table 4:** GO term annotations of genes significantly up-regulated in the 59A sch9-deleted mutant.

GO category	GO no.	P*-*value	GO description	k	f
Molecular function	GO:0016491	1.999e-06	oxidoreductase activity	19	272
	GO:0050839	8.175e-06	cell adhesion molecule binding	3	3
Biological process	GO:0007155	6.496e-08	cell adhesion	5	7
Biological process	GO:0000755	1.704e-07	cytogamy	5	8
	GO:0000746	7.986e-07	conjugation	4	5
	GO:0055114	1.999e-06	oxidation-reduction process	19	272
	GO:0006950	1.094e-05	response to stress	13	152
Cellular component	GO:0005576	5.151e-10	extracellular region	15	95
	GO:0005886	5.299e-09	plasma membrane	26	350
	GO:0001950	1.36e-08	plasma membrane enriched fraction	13	86
	GO:0009277	9.462e-07	fungal-type cell wall	11	85
	GO:0005618	7.976e-06	cell wall	9	68
	GO:0031225	2.78e-05	anchored to membrane	8	61
	GO:0000015	7.934e-05	phosphopyruvate hydratase complex	3	5

k: number of genes in the family identified as affected in the experiment; f: total number of genes in the family.

### Is the stress response an adaptation to nitrogen/lipid imbalance?

**Figure 7:**
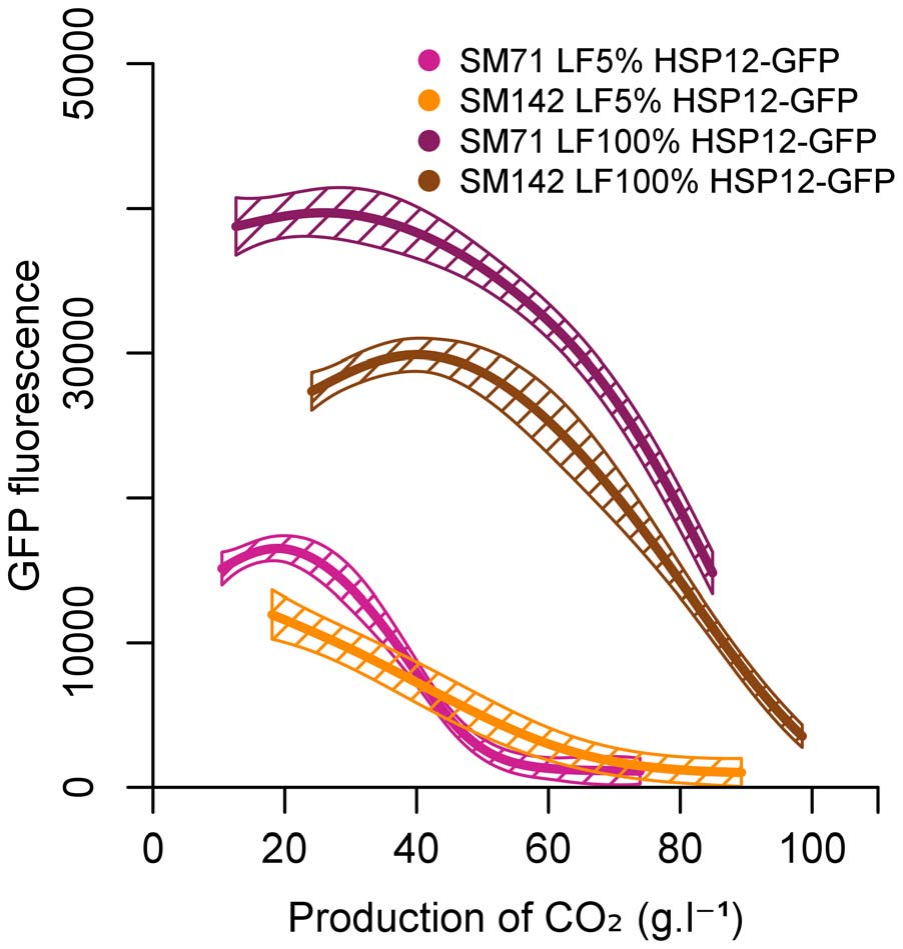
Effect of the nutrient imbalances on HSP12 stress gene expression in *S. cerevisiae* EC1118 during alcoholic fermentation. The synthetic medium contained 71 mg/L (SM71) or 142 mg/L (SM142) assimilable nitrogen, and 5% or 100% lipid factors (LF5% or LF100%). Fluorescence of the GFP reporter protein, produced under the control of the HSP12 promoter, was measured by flow cytometry. The graphs are the result of smoothing of measurements series (at least 3 repetitions) using the software R. Smoothing obtained is framed by a confidence interval calculated at 95%.

Since *HSP12* was strongly overexpressed in the sch9-deleted strain and was shown to play a role in cell protection, we aimed to monitor its response in our conditions. We constructed a derivative of the strain EC1118 producing *GFP* under the control of the *HSP12* promoter. We checked that this *hsp12::GFP* strain had a rate of CO_2_ production (Figure S4), a cell population (data not shown) and a viability (Figure S5) similar to the native strain during fermentation in each SM142 LF5% and SM71 LF5%. Then we used flow cytometry to monitor the activity of the *HSP12* gene promoter, during fermentation under various conditions of nutritional imbalance (Figure 7). The *HSP12*-gene promoter was mainly active at the beginning of the stationary phase, decreasing thereafter in all the nutrient conditions tested: its activity was independent of nitrogen source, but depended on the lipids in the medium, as previously observed by Rossignol [27]. In our conditions, cells with greater viability had higher *HSP12* gene promoter activity, consistent with the protective function of *HSP12* under stress conditions [28]. However, deletion of the *HSP12* gene has no effect on the mortality of the wine yeasts when both lipid and nitrogen were limited (data not shown). In our study, the absence of phenotype for *HSP12* deletion is in line with several other papers that show no phenotype for deletion or overexpression of this gene under other stress conditions [29], [30].

## Discussion

Alcoholic fermentations of white and some rosé wines take place after clarification steps which decrease the lipid content of the must. Alcoholic fermentations are performed in conditions of strong anaerobiosis which prevents biosynthesis of sterols and unsaturated fatty acids. Several studies have reported the negative effect on wine fermentations of excessive clarifications of musts [6], [31], [32]. They lead to sluggish fermentations associated with high rates of yeast cell death and often to stuck fermentations. Yeast cell death in such conditions has been considered to be the direct outcome of membrane lipid changes and the inability of yeast to cope with ethanol toxicity. Indeed, we observed in our experiments that ergosterol availability had a major effect on yeast viability during fermentation, consistent with the findings of Luparia et al. [7]. The protective effect of ergosterol may be related to a substantial restoration of the rigidity of the plasma membrane [33], ergosterol being the main sterol in yeast: it plays important roles in the construction and maintenance of membrane structures and properties, such as integrity, fluidity, permeability, ethanol resistance and H^+^-ATPase activity (for a review, see [9]). The relevance of other nutrients on yeast cell death in such conditions has not previously been considered. Here, we show that yeast cell death in lipid-limited fermentations is strongly modulated by the nitrogen content of the medium, with high nitrogen availability leading to high rates of cell death. The triggering of yeast cell death was not restricted to a particular nitrogen source but was caused by various sources, including amino acids and ammonium, albeit to different extents. When included in SM71, only those amino acids that are metabolised were toxic: arginine, glutamate and glutamine caused cell death whereas proline and histidine did not. This suggests that the uptake of amino acids is required for this toxicity. We show that this toxic effect is dependent on the TOR nitrogen signalling pathway, and inhibition of the TOR kinase by rapamycin increased viability. The TOR kinase TORC1 is involved in sensing the nitrogen status of the cell and controls various cellular processes in response to nitrogen [34]. We also found that deletion of the gene encoding the TOR-associated protein Tco89, member of the TORC1 multiprotein complex required for TOR signalling, restored high viability in lipid-limited fermentations, providing further evidence of TOR involvement (data not shown). Our data therefore implicate TOR nitrogen signalling in the triggering of yeast cell death in lipid-limited fermentations. We report the first evidence of a relationship between the TORC1 nitrogen signalling pathway and lipid limitation in alcoholic fermentations. The involvement of nitrogen in wine yeast cell death has been recently reported in other situations [15]–[17], [35], for example in aging yeast cells. Ammonium is toxic for yeast and TOR signalling appears to be involved, but SCH9 deletion decreased yeast survival in these conditions [17]: the mechanism of the phenomenon we report is likely to be different because *SCH9* deletion improved yeast cell viability in our model. Most studies have analysed cell death as a chronological ageing process, dependent on time [21], [22], [35], [36]. Here, we show that cell death during fermentation was controlled by the progress of fermentation, i.e. correlated with the ethanol content of the medium, consistent with a central role for ethanol.

Our experiments reveal a previously unsuspected role for nitrogen sources in wine fermentation: they have toxic effects when associated with low availability of lipids and specifically sterols. The role of nitrogen in cell death is obviously complex because the highest nitrogen levels were not found to be as toxic as intermediate concentrations. Many residual amino acids were detected in the medium in these conditions suggesting that they may act through other sensing pathways that interact with the TOR signalling outputs. The apparent complexity of the effects of nitrogen sources has probably contributed to obscuring their role in triggering yeast cell death, because high nitrogen levels are not always necessarily associated with high cell death rates in lipid-limited musts. Our data provide a basis for further analysis of the mechanisms and conditions which favour nitrogen toxicity in wine alcoholic fermentations.

Sch9p is a serine/threonine protein kinase localised at the vacuole surface; it is a target of TORC1 and is central to nutrient-mediated signalling [19]. TORC1-independent functions of Sch9p, with a direct link between Sch9p and nitrogen metabolism, have also been suggested [22]. In addition, Sch9p integrates also stress signal from sphingolipids, whose synthesis was related to lifespan in yeast [37]. But in our conditions, only limited changes in expression of genes involved in sphingolipid biosynthesis were observed. In fact, we found 6 genes related to sphingolipid biosynthesis, metabolism or signaling whose expression was down-regulated in the *SCH9* mutant vs wt. However, they have a log FC between −0.86 and −0.39 and only genes with log2 fold change greater than 1 (positive or negative) were considered. We show that *SCH9* is involved in the control of cell death in alcoholic fermentation conditions. Our transcriptional analysis of a *SCH9*-deleted strain suggests that Sch9p may act through a modulation of the expression of many genes in our conditions. Indeed, several genes whose expression is controlled by Sch9p are expected to play a role in yeast cell protection and may contribute to cell survival. The deletion of *SCH9* did not, however, exactly recapitulate the effect of nitrogen source modulation as exemplified by the response of *HSP12* which is strongly regulated by *SCH9* deletion but only weakly by nitrogen availability. In addition, the response of *HSP12* highlights the existence of specific control of the expression of a stress gene by lipids and in particular down-regulation of its expression by low lipid availability.

The overall stress response during fermentation, as evaluated from the storage glycogen and trehalose, was higher when yeast cells were starved of nitrogen than when starved of ergosterol in lipid-limited-fermentations. Yeast cells are obviously unable to elicit an appropriate stress response when growth is stopped by limiting sterol availability. Such limitations are probably uncommon in natural environments since only trace amounts (10 mg/L) of oxygen permit the production of sufficient sterols [38]. Therefore, the conditions encountered by yeasts in wine alcoholic fermentations are extreme conditions for which yeasts have not developed an adequate adaptation system. Indeed, our experimental model may be considered to impose nutrient restrictions unlikely to be encountered in nature and can be related to auxotrophic mutants that die rapidly upon starvation [10]: yeast is auxotrophic for sterols and unsaturated fatty acids when no oxygen is available.

Our data show, for the first time, that nitrogen excess in situation of low lipid, and low oxygen availability, as is common in wine making processes, may lead to yeast cell death and potentially to stuck fermentations. This work provides new notions to consider when defining nutrient management strategies for wine alcoholic fermentations. Nitrogen has been considered to be a positive factor in the outcome of alcoholic fermentations and nitrogen (in ammonium form) addition to musts or fermenting wines is a common practice. We demonstrate that nitrogen sources can potentially have a negative impact due to their signalling effects and this need to be taken into account. An in-depth understanding of the specific effects of each nitrogen source under such conditions is required to improve the prediction of the risks associated with nitrogen excess in lipid-limited fermentations.

## Materials and Methods

### Yeast strains

Lalvin EC1118 is a *S. cerevisiae* wine yeast isolated in Champagne (France) and manufactured by Lallemand (Montreal, Canada): 59A is a strain generated from a meiotic haploid spore isolated from Lalvin EC1118, and selected for its similar fermentation performance and metabolite production [39]. The EC1118 strain with *HSP12* promoter-driven GFP expression was constructed by inserting the GFP coding sequence at the 3′-end of the *HSP12* promoter by standard homologous recombination with a yEGFP-KanMX cassette amplified from pKT127 (pFA6a-link-yEGFP-KAN, P30175 Euroscarf strain) using synthetic primers (1A and 1B in Table 5). A SCH9-deleted mutant of 59A was generated by PCR-mediated gene disruption using the *loxP-KanMX-loxP* cassette of the pUG6 vector amplified with synthetic primers (2A and 2B in Table 5), to replace the ORF in 59A strain with a gene that confers resistance to G418. Gene disruptions and constructs were confirmed by PCR (primers 1C and 1D for EC1118 *hsp12::GFP*, and 2C to 2F for 59A *sch9::KanMX*, respectively, Table 5).

**Table 5:** Synthetic oligonucleotides.

Name	Sequence
1A	AACTCAAAACAAAAAAAACTAAATACAACAATGTCTGACGCAGGTGACGGTGCTGGTTTA
1B	AGAAAAAACCATGTAACTACAAAGAGTTCCGAAAGATTCGATGAATTCGAGCTCG
1C	GTGGAGTGCGATTTGTTCGT
1D	CCAACCAACGCATCAAGAGA
2A	TTAGCTCTCAACACCAACATCCAAATGGACAGAACATTCGTACGCTGCAGGTCGAC
2B	CTTCCACTGACAAATTCGTCATCCATGTGTTGGTCGCATAGGCCACTAGTGGATCTG
2C	ATCGTCGAATCAGGATACTGGA
2D	CAAGAGGAGCGATTGAGAAA
2E	ATTACGGCTCCTCGCTGCAG
2F	TGATTTTGATGACGAGCGTAAT

### Synthetic culture media

Unless otherwise specified, synthetic fermentation medium with 142 mg/L assimilable nitrogen (SM142) and 23% glucose + fructose (1/1), strongly buffered to pH 3.3 and simulating one third nitrogen and amino acid concentrations of a standard grape juice [40] was routinely used. This medium contained, per litre: 115 g glucose, 115 g fructose, 6 g citric acid, 6 g DL-malic acid, 750 mg KH_2_PO_4_, 500 mg K_2_SO_4_, 250 mg MgSO_4_.7H_2_O, 155 mg CaCl_2_.2H_2_O, 200 mg NaCl, 4 mg MnSO_4_.H_2_O, 4 mg ZnSO_4_. 7H_2_O, 1 mg CuSO_4_.5H_2_O, 1 mg KI, 0.4 mg CoCl_2_.6H_2_O, 1 mg H_3_BO_3_, 1 mg (NH_4_)_6_Mo_7_O_24_, 20 mg myo-inositol, 2 mg nicotinic acid, 1.5 mg calcium panthotenate, 0.25 mg thiamine- HCl, 0.25 mg pyridoxine and 0.003 mg biotin. It also contained ammoniacal nitrogen and amino acids as nitrogen sources (per litre): 153 mg NH_4_Cl, 204 mg L-proline, 169 mg L-glutamine, 60 mg L-tryptophane, 48 mg L-alanine, 40 mg L-glutamic acid, 26 mg L-serine, 25 mg L-threonine, 16 mg L-leucine, 15 mg L-aspartic acid, 15 mg L-valine, 13 mg L-phenylalanine, 125 mg L-arginine, 11 mg L-histidine, 11 mg L-isoleucine, 10 mg L-methionine, 6 mg L-glycine, 6 mg L-lysine, 6 mg L-tyrosine and 4 mg L-cysteine. The medium was heat-sterilized (100°C, 10 min). Lipid factors (LF) were added to the medium after sterilization. The LF final concentration in the fermentation medium (LF 100%) was 4.5 mg/L oleic acid and 15 mg/L ergosterol. To evaluate the adaptation to nutrient limitations, media containing different concentrations of nitrogen sources (SM142 and SM71) with two different LF contents (100% or 5%) were tested.

### Fermentation conditions and kinetics

The yeast strains used in this study were precultured in a nutrient medium containing Yeast Nitrogen Base (YNB) without amino acids (6.7 g/L) and glucose (20 g/L), for 24 h at 28°C in Erlenmeyer flasks. The fermentation medium was inoculated with 1 10^6^ cell/mL from preculture. Yeast cultures were carried out in fermenters (1.2 L, containing 1 L medium), with fermentation locks (CO_2_ bubbling outlets filled with water). Fermentation media were routinely de-aerated prior to inoculation by bubbling pure argon for 5 min. Filling conditions were controlled and fermentations were carried out under anaerobic and isothermal conditions (24°C), with permanent stirring (300 rpm).

The amount of CO_2_ released was calculated from automatic measurements (taken every 20 min) of fermenter weight [41]. The CO_2_ production rate was calculated by polynomial smoothing.

### Establishment of the fermentation conditions

The aim of this study was to determine how *S. cerevisiae* wine yeast strains respond to lipid limitation in the presence of various nitrogen contents. This was done by monitoring CO_2_ production, cell counts and yeast viability in a synthetic grape juice mimicking an oenological environment. Two main fermentation conditions, with 230 g/L glucose + fructose (1/1), were established with different LF concentrations in the culture medium (LF100% and LF5%): SM142 and SM71 contain, respectively, one third and one sixth of the nitrogen concentration required for the yeast strain to complete fermentation. These N-limiting conditions were chosen to ensure sluggish fermentations. For rapamycin treatment, cells were grown in SM142 with LF5% and 20 nM rapamycin was added at T0. The conditions of growth of wt and sch9 strains used for microarray analysis were SM71 and SM142 with LF 5%.

### Cell population densities, cell viability and GFP activity determinations

Cell population densities, cell viability and Hsp12 promoter activity were determined by flow cytometry using a C6 cytometer (Accuri, BD Biosciences). To determine cell population densities, the cell suspension was compared to a suspension of latex beads of known concentration. For cell viability analysis, propidium iodide (PI) (Calbiochem) was added to the cell suspension (5 µL of 0.1 mg/mL solution), and the samples mixed by gentle shaking. PI is a fluorescent nucleic acid stain (excitation 488 nm, emission 575 nm) which cannot penetrate intact cell membranes. PI flow cytometry analysis was performed 10 min after staining. Fluorescence data for cells stained by PI were collected in channel FL3. Viability was determined as the percentage of intact and fragile cells among all cells [42]. For Hsp12 promoter activity, GFP fluorescence (excitation 488 nm, emission 530 nm) was collected in channel FL2.

### Determination of the trehalose and glycogen contents

Trehalose was extracted from cells with 0.5 M trichloracetic acid (TCA) and quantified with anthrone according to Rossignol [27]. Glycogen was extracted from the same sample with HCl-DMSO and treated with an amyloglucosidase enzyme. The glucose formed was assayed by colorimetry [43]. Results are expressed as % dry weight.

### RNA extraction and DNA microarray analyses

Total RNAs were isolated from wild type 59A cells and sch9-deleted mutant cultures at 35 g of CO_2_ production, by the TRIzol® method according to Chomczynski and Sacchi [44]. Aliquots of 10^9^ cells were harvested and quickly washed with 750 µL cooled (4°C) DEPC-treated water. Cells were pelleted and frozen in a −80°C methanol bath. Frozen cells were mechanically lysed by vortexing with glass beads (d = 0.3 mm) in 400 µL TRIzol® (GIBCO BRL) at 4°C for 15 min. The liquid phase was collected and TRIzol® added to give a final volume of 4 mL. The samples were mixed and incubated for 5 min at room temperature, and 800 µL chloroform was added. The mixture was vortexed and then incubated for 3 min and centrifuged (9,000 *g* for 15 min). The supernatant was centrifuged again (2,000 *g* for 2 min) in swing buckets. RNAs were pelleted from 2 mL aliquots of the supernatant by the addition of 2 mL cooled isopropanol (−20°C) and incubated for 10 min. The samples were centrifuged (9,000 *g* for 10 min) and the resulting nucleic acid pellet was washed twice with 750 µL 75% ethanol/DEPC-treated water and then dissolved in 150 µL of nuclease-free water (Qiagen).

Total RNA from 100 µg aliquots of these preparations was purified with a RNeasy® mini kit (Qiagen) following the RNA cleanup protocol, including membrane DNase digestion. RNAs were eluted with 2 × 30 µL of the provided RNAse-free water. RNA quality was verified by capillary electrophoresis with an RNA 6000 Nano LabChip Kit (Agilent Technologies). Samples of 100 ng of purified RNA were labelled with Low input Quick Amp Labelling one-colour kit (Agilent Technologies) according to manufacturer's recommendation (indirect labelling of mRNAs with Cyanin 3 dCTP dye). RNAs were hybridised on 8 × 15 k array Agilent standard Yeast V2 Gene Expression Microarrays (Agilent Technologies) for 17 h in a rotating oven at 65°C following the manufacturer's recommendation. A Genepix 4000B scanner was used for array digitalization: the laser voltage was set to avoid signal saturation and data was extracted with GenePix® Pro 7 software (Molecular Devices).

R.2.14.21. was used for statistical analysis [45]. The limma package [46]–[48] was used to normalise the microarray data (quantile method for normalisation between arrays). To analyse differential gene expression between experimental conditions, a modified t-test was used by filtering on confidence at p<0.05, using the Benjamini and Hochberg false discovery rate as multiple testing corrections of the t-test p-values [49]. Only genes with a log2 fold change greater than 1 (positive or negative) were considered. The complete data set is available through the Gene Expression Omnibus database (accession number GSE42027). For a statistical treatment of groups of genes, data were analysed using the web-based tool Funspec (http://funspec.med.utoronto.ca/; [50]) (P-value < 0.05 and Bonferroni correction) and genes were classified into functional categories, biological process and protein cellular localisations using the GO Database.

## Supporting Information

Figure S1 **Effect of nutrient imbalances on the cell population of** ***S. cerevisiae*** **EC1118 cultures during alcoholic fermentation at 24°C according to CO_2_ production.** The synthetic medium contained 71 mg/L (SM71), 142 mg/L (SM142) or 425 mg/L (SM425) assimilable nitrogen, and 5% or 100% lipid factors (LF 5% or LF100%). The graphs are the result of smoothing of measurement series (at least 3 repetitions) using the software R.
(TIFF)

Figure S2 **Effect of lipid factors on the rate of CO_2_ production of** ***S. cerevisiae*** **EC1118 cultures during alcoholic fermentation at 24°C.** The synthetic medium contained 142 mg/L assimilable nitrogen (SM142) and 5% lipid factor (LF5%), with or without additional oleic acid or ergosterol (content as in LF 100%). The graphs are the result of smoothing of measurement series (at least 3 repetitions) using the software R.
(TIFF)

Figure S3 **Effect of nitrogen sources on the rate of CO_2_ production of** ***S. cerevisiae*** **EC1118 cells during alcoholic fermentation at 24°C.** The synthetic medium contained 71 mg/L assimilable nitrogen (SM71) and 5% lipid factors (LF5%), with or without additional arginine, glutamine, glutamate, histidine or proline (content as in SM142). The graphs are the result of smoothing of measurement series (at least 3 repetitions) using the software R.
(TIFF)

Figure S4 **Effect of nutrient imbalances on the rate of CO_2_ production by cultures of** ***S. cerevisiae*** **EC1118 carrying the HSP12-GFP fusion during alcoholic fermentation at 24°C.** The synthetic medium contained 71 mg/L (SM71) or 142 mg/L (SM142) assimilable nitrogen, and 5% or 100% lipid factors (LF5% or LF100%). The graphs are the result of smoothing of measurement series (at least 3 repetitions) using the software R.
(TIFF)

Figure S5 **Effect of nutrient imbalances on the viability of** ***S. cerevisiae*** **EC1118 carrying the HSP12-GFP fusion during alcoholic fermentation at 24°C.** The synthetic medium contained 71 mg/L (SM71) or 142 mg/L (SM142) assimilable nitrogen, and 5% lipid factors (LF5%). The graphs are the result of smoothing of measurement series (at least 3 repetitions) using the software R.
(TIFF)

Table S1 **Effect of nutrient imbalances on the amino acid content of** ***S. cerevisiae*** **EC1118 cells (µmol assimilable nitrogen/L).**
(DOC)

Table S2 **Genes that were significantly down-regulated in the 59A** ***SCH9*** **-deleted mutant.**
(DOC)

Table S3 **Genes that were significantly up-regulated in the 59A** ***SCH9*** **-deleted mutant.**
(DOC)
