# Supplementary figures and images for: Impact of Nutrient Imbalance on Wine Alcoholic Fermentations: Nitrogen Excess Enhances Yeast Cell Death in Lipid-Limited Must
Source: PLoS One. 2013 Apr 26;8(4):e61645. doi: 10.1371/journal.pone.0061645 (PMC3637302; doi:10.1371/journal.pone.0061645)

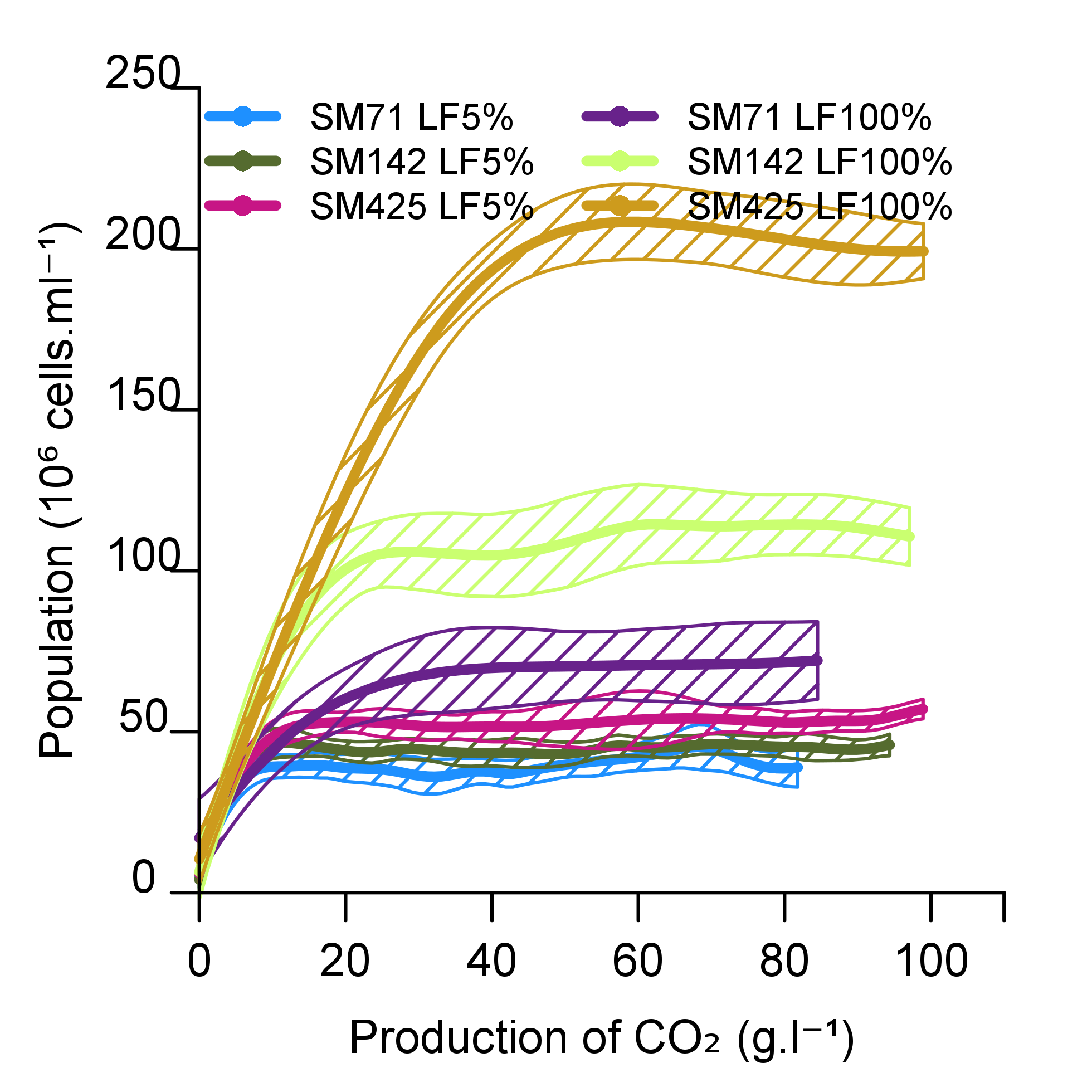

Supplement: Figure S1 — Effect of nutrient imbalances on the cell population of S. cerevisiae EC1118 cultures during alcoholic fermentation at 24°C according to CO2 production. The synthetic medium contained 71 mg/L (SM71), 142 mg/L (SM142) or 425 mg/L (SM425) assimilable nitrogen, and 5% or 100% lipid factors (LF 5% or LF100%). The graphs are the result of smoothing of measurement series (at least 3 repetitions) using the software R. (TIFF) [file pone.0061645.s001.tiff]

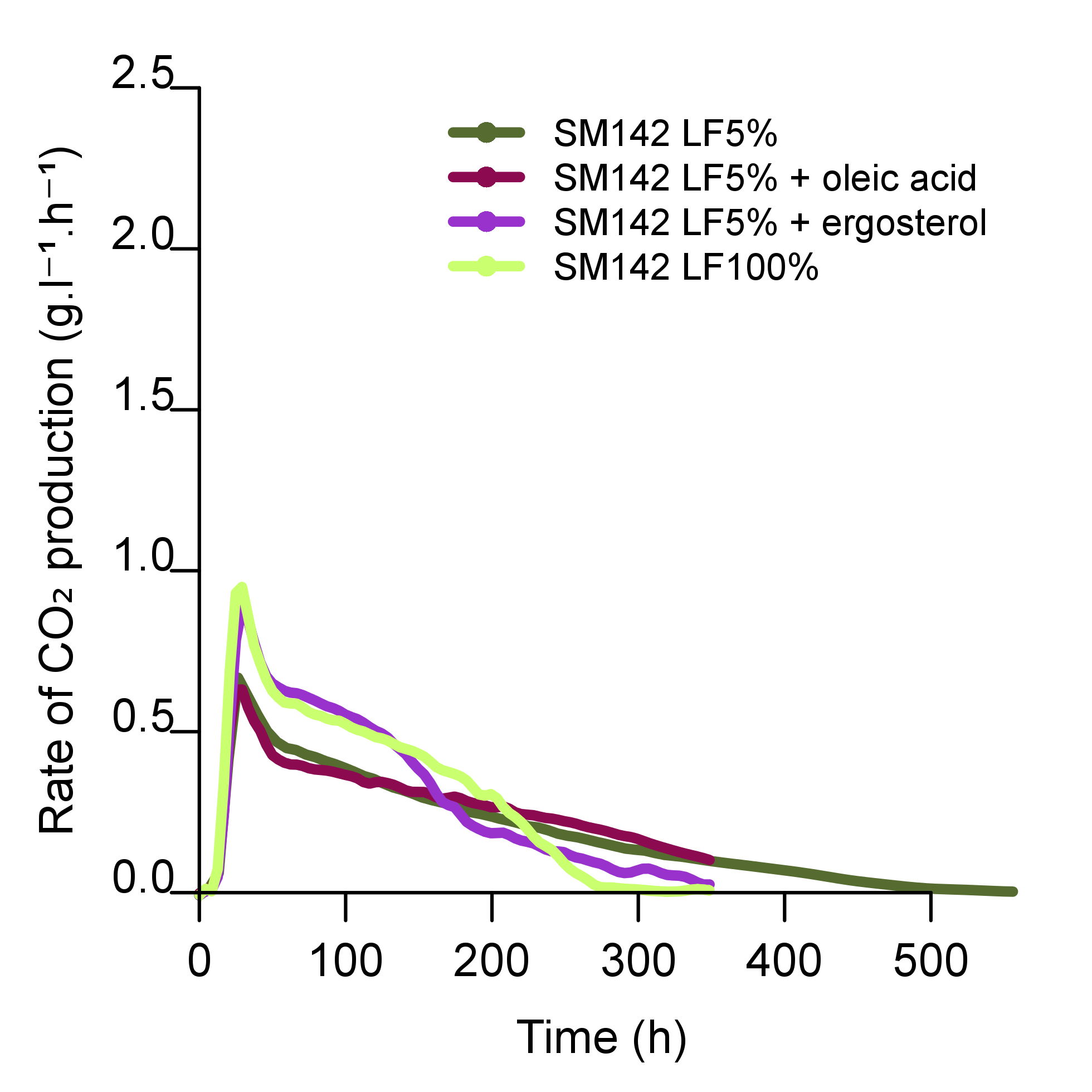

Supplement: Figure S2 — Effect of lipid factors on the rate of CO2 production of S. cerevisiae EC1118 cultures during alcoholic fermentation at 24°C. The synthetic medium contained 142 mg/L assimilable nitrogen (SM142) and 5% lipid factor (LF5%), with or without additional oleic acid or ergosterol (content as in LF 100%). The graphs are the result of smoothing of measurement series (at least 3 repetitions) using the software R. (TIFF) [file pone.0061645.s002.tiff]

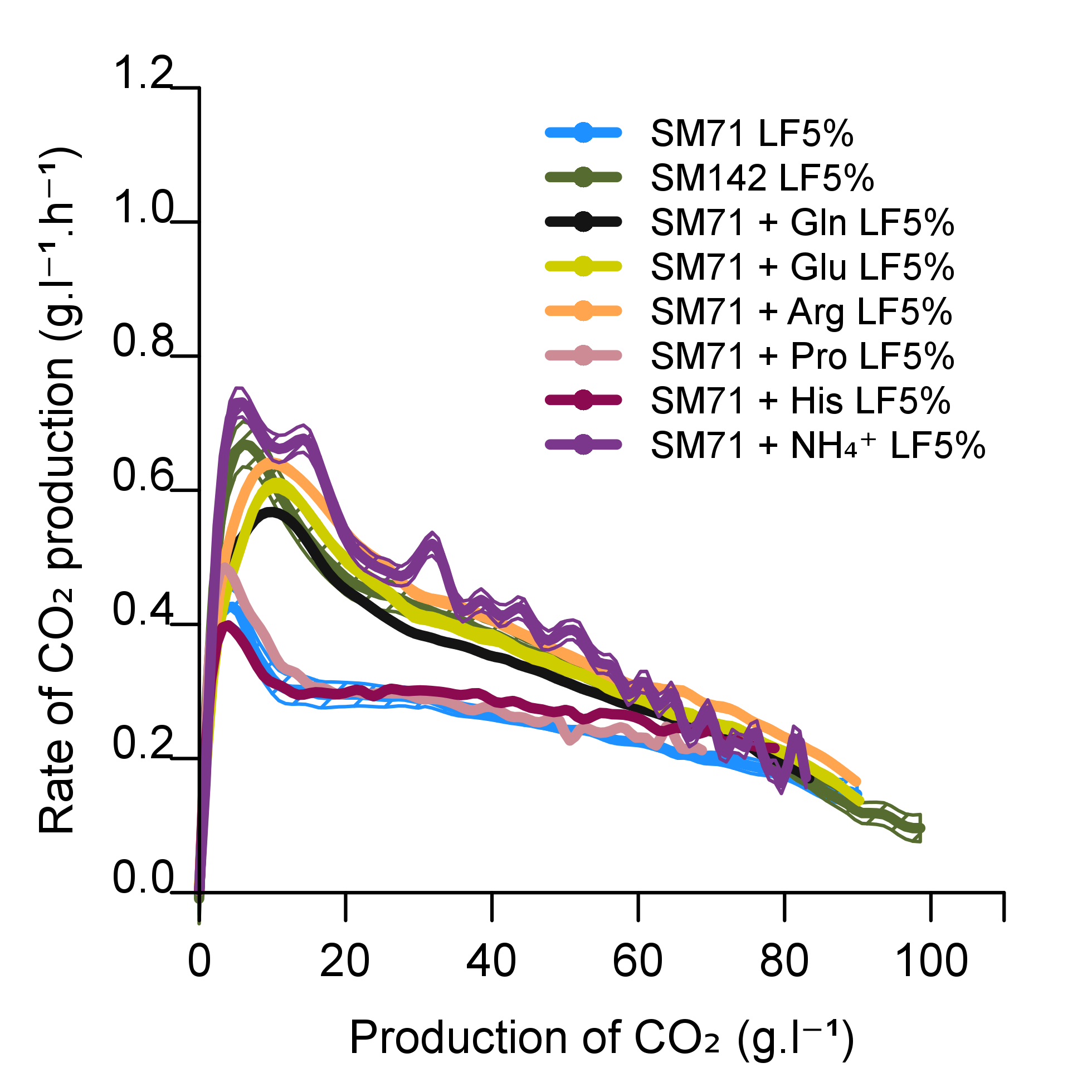

Supplement: Figure S3 — Effect of nitrogen sources on the rate of CO2 production of S. cerevisiae EC1118 cells during alcoholic fermentation at 24°C. The synthetic medium contained 71 mg/L assimilable nitrogen (SM71) and 5% lipid factors (LF5%), with or without additional arginine, glutamine, glutamate, histidine or proline (content as in SM142). The graphs are the result of smoothing of measurement series (at least 3 repetitions) using the software R. (TIFF) [file pone.0061645.s003.tiff]

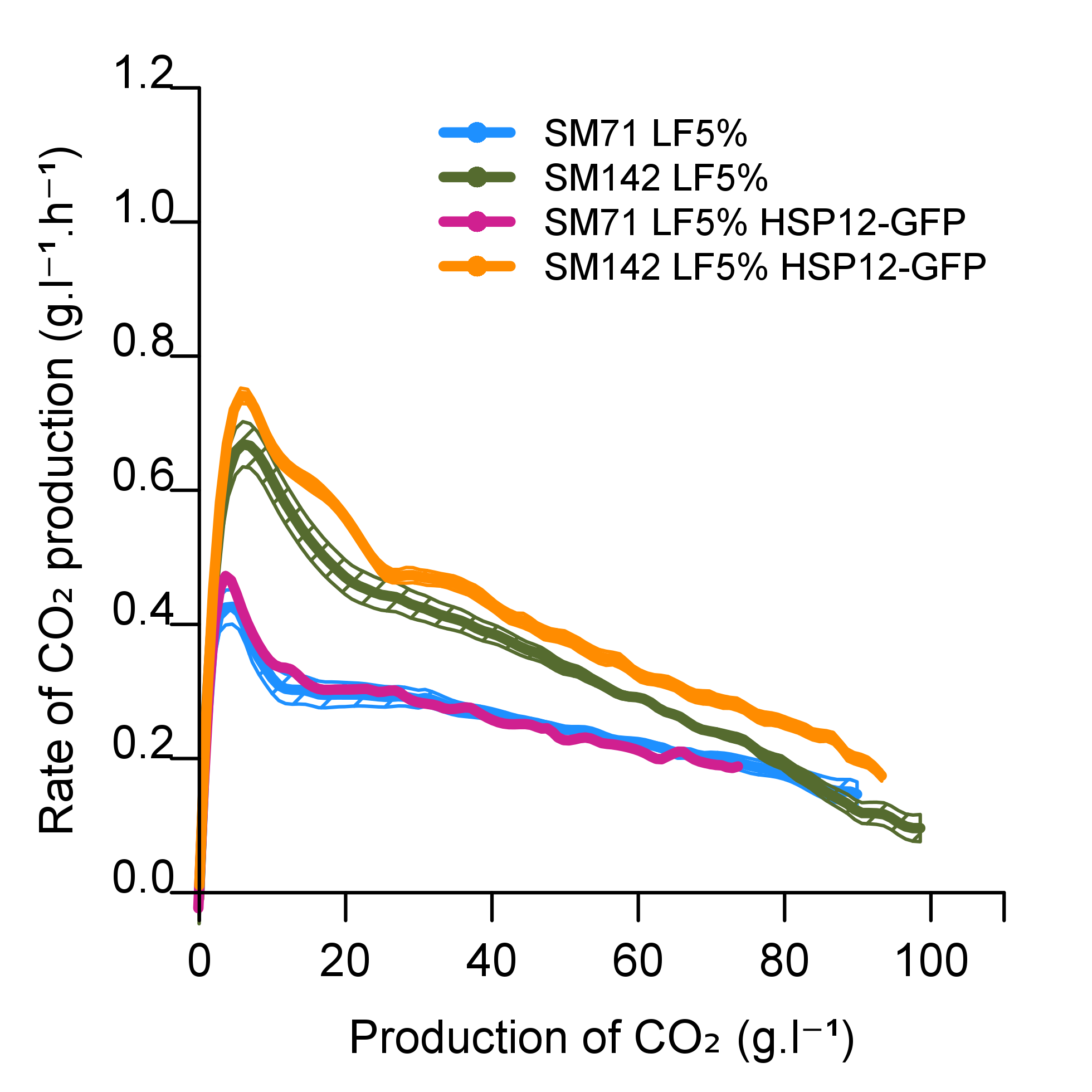

Supplement: Figure S4 — Effect of nutrient imbalances on the rate of CO2 production by cultures of S. cerevisiae EC1118 carrying the HSP12-GFP fusion during alcoholic fermentation at 24°C. The synthetic medium contained 71 mg/L (SM71) or 142 mg/L (SM142) assimilable nitrogen, and 5% or 100% lipid factors (LF5% or LF100%). The graphs are the result of smoothing of measurement series (at least 3 repetitions) using the software R. (TIFF) [file pone.0061645.s004.tiff]

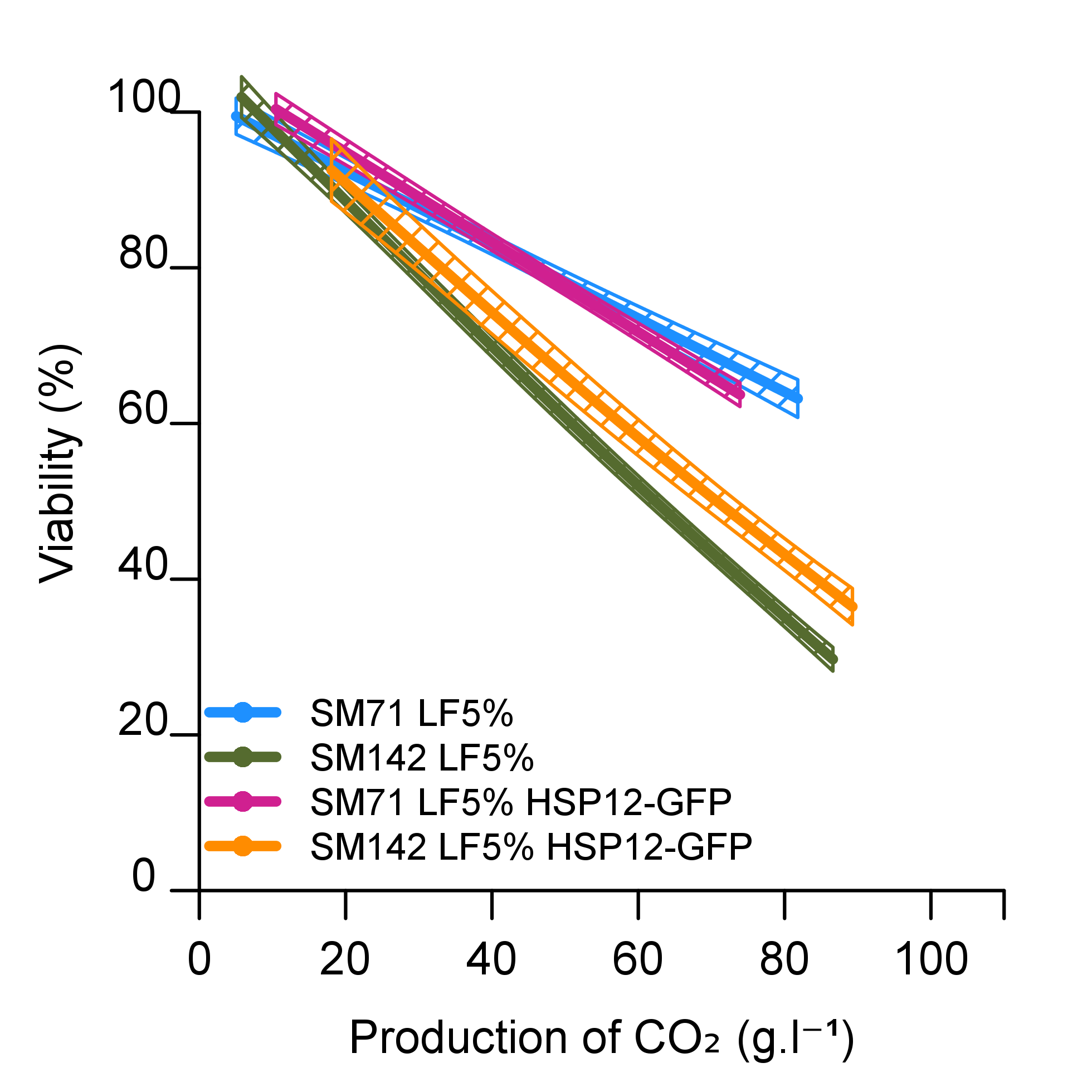

Supplement: Figure S5 — Effect of nutrient imbalances on the viability of S. cerevisiae EC1118 carrying the HSP12-GFP fusion during alcoholic fermentation at 24°C. The synthetic medium contained 71 mg/L (SM71) or 142 mg/L (SM142) assimilable nitrogen, and 5% lipid factors (LF5%). The graphs are the result of smoothing of measurement series (at least 3 repetitions) using the software R. (TIFF) [file pone.0061645.s005.tiff]
