# Supplementary material for: Impact of Nutrient Imbalance on Wine Alcoholic Fermentations: Nitrogen Excess Enhances Yeast Cell Death in Lipid-Limited Must
Source: PLoS One. 2013 Apr 26;8(4):e61645. doi: 10.1371/journal.pone.0061645 (PMC3637302; doi:10.1371/journal.pone.0061645)
Supplement: Table S1 — Effect of nutrient imbalances on the amino acid content of S. cerevisiae EC1118 cells (µmol assimilable nitrogen/L). (DOC) [file pone.0061645.s006.doc]

**Table S1 Effect of nutrient imbalances on the amino acid content of *S. cerevisiae* EC1118 cells** (µmol assimilable nitrogen/L).

|  |  |  |  |  |  |  |  |  |  |  |  |  |  |  |  |
| --- | --- | --- | --- | --- | --- | --- | --- | --- | --- | --- | --- | --- | --- | --- | --- |
|  |  |  | **SM71 LF5%** |  |  |  |  | **SM142 LF5%** |  |  |  |  | **SM425 LF5%** |  |  |
| **time (h)** | **0** | **24** | **48** | **72** | **238** | **0** | **24** | **48** | **72** | **216** | **0** | **24** | **48** | **72** | **216** |
| **g of CO_2_ produced** | **0** | **5** | **15** | **24** | **74** | **0** | **5.8** | **19.4** | **31.9** | **78.9** | **0** | **4.55** | **22.2** | **36.6** | **80.8** |
| Alanine | 273.6 | 0 | 0 | 0 | 0 | 516.9 | 154.6 | 0.9 | 0 | 15.1 | 1640.45 | 1473.65 | 1435.75 | 1458.3 | 1695.25 |
| Arginine | 1395.6 | 0 | 0 | 0 | 0 | 2691.2 | 520 | 0 | 0 | 0 | 8515.4 | 6722.4 | 6407 | 6416.4 | 6643.6 |
| Aspartic acid | 47.5 | 1.2 | 1.4 | 1.2 | 2.4 | 84.1 | 5.7 | 2 | 3.3 | 9.6 | 308.45 | 207.35 | 189.2 | 188.7 | 203.7 |
| Cystine | 16 | 2.8 | 4.4 | 3.6 | 2.2 | 23 | 24.2 | 26.8 | 21 | 21.8 | 0 | 0 | 0.3 | 0.3 | 1.2 |
| Glutamine | 624.6 | 0 | 0 | 0 | 0 | 1026.2 | 155.4 | 0 | 0 | 11.6 | 3625.3 | 2122.9 | 1857.9 | 1839.9 | 1992.3 |
| Glutamic acid | 134.9 | 0 | 0 | 0 | 0 | 268.8 | 44 | 0 | 0 | 6.5 | 787.85 | 630.35 | 586.5 | 575.6 | 614.2 |
| Glycine | 40.7 | 0 | 0 | 0 | 2.5 | 76 | 66.1 | 7.4 | 0 | 23.5 | 240.15 | 230.65 | 233.1 | 238.75 | 275.75 |
| Histidine | 91.5 | 0 | 0 | 0.6 | 0 | 180.9 | 0 | 0 | 0 | 0 | 567.6 | 257.25 | 128.55 | 72.9 | 83.1 |
| Isoleucine | 40.8 | 0 | 0.1 | 0 | 0 | 79.1 | 0 | 0 | 0 | 0 | 250.45 | 125.85 | 101.55 | 96.75 | 100.65 |
| Leucine | 66.7 | 0 | 0 | 0 | 0 | 120.7 | 5.7 | 1.5 | 8 | 13.2 | 375.3 | 120.95 | 96.85 | 91.5 | 99.8 |
| Lysine | 34.4 | 0 | 0 | 0 | 0 | 67.6 | 0 | 0 | 0 | 2.6 | 205.1 | 0 | 0 | 0 | 11.1 |
| Methionine | 27.6 | 0 | 0 | 0 | 0 | 54 | 0 | 0 | 0 | 0 | 183.75 | 56.45 | 42.7 | 41.6 | 42.85 |
| Phenylalanine | 38.6 | 0 | 0 | 0 | 0 | 73.4 | 2.9 | 0 | 0 | 10.2 | 232.45 | 123.65 | 106.65 | 104.1 | 114.3 |
| Serine | 123.7 | 0 | 0 | 0 | 0 | 232.4 | 9.6 | 1.3 | 0 | 4.5 | 732 | 431.8 | 360.25 | 354.45 | 370.3 |
| Threonine | 111 | 0 | 0 | 0 | 0 | 209.1 | 5.3 | 0.6 | 0 | 0 | 653.9 | 302 | 231.95 | 227 | 235.85 |
| Tryptrophan | 375.8 | 0 | 0 | 0 | 0 | 744.6 | 437.4 | 0 | 0 | 0 | 2373.9 | 1769.1 | 1322.9 | 1089.1 | 669.2 |
| Tyrosine | 17.9 | 0 | 0 | 0 | 0 | 32.6 | 16.7 | 0 | 0 | 1.6 | 103.05 | 89.15 | 83.85 | 83.55 | 86.65 |
| Valine | 63.5 | 0 | 0 | 0 | 0 | 120.3 | 12.4 | 0 | 0 | 0 | 383.7 | 278.8 | 262.25 | 266.2 | 295.95 |
| **Sum without proline** | **3524.4** | **4** | **5.9** | **5.4** | **7.1** | **6600.9** | **1460** | **40.5** | **32.3** | **120.2** | **21178.8** | **14942.3** | **13447.25** | **13145.1** | **13535.75** |
| Proline | 940.3 | 975.4 | 1006.2 | 988.8 | 981.9 | 1637.5 | 1647.4 | 1806.7 | 1874.6 | 1888 | 5251.1 | 4860.2 | 4811.65 | 4860.85 | 4954.45 |
| Sum with proline | 4464.7 | 979.4 | 1012.1 | 994.2 | 989 | 8238.4 | 3107.4 | 1847.2 | 1906.9 | 2008.2 | 26429.9 | 19802.5 | 18258.9 | 18005.95 | 18490.2 |
