# Supplementary material for: Impact of Nutrient Imbalance on Wine Alcoholic Fermentations: Nitrogen Excess Enhances Yeast Cell Death in Lipid-Limited Must
Source: PLoS One. 2013 Apr 26;8(4):e61645. doi: 10.1371/journal.pone.0061645 (PMC3637302; doi:10.1371/journal.pone.0061645)
Supplement: Table S2 — Genes that were significantly down-regulated in the 59A SCH9 -deleted mutant. (DOC) [file pone.0061645.s007.doc]

**Table S2 Genes that were significantly down-regulated in the 59A *SCH9*-deleted mutant.**

| Systematic name | Common name | logFC | *P* value | SGD Annotation |
| --- | --- | --- | --- | --- |
| YHR205W | SCH9 | -2.95 | 1.29e-08 | Protein kinase involved in transcriptional activation of osmostress-responsive genes; regulates G1 progression, cAPK activity, nitrogen activation of the FGM pathway; involved in life span regulation; homologous to mammalian Akt/PKB |
| YGR190C | . | -2.75 | 5.23e-07 | Dubious open reading frame unlikely to encode a protein, based on available experimental and comparative sequence data; overlaps the verified gene HIP1/YGR191W |
| YNL231C | PDR16 | -2.51 | 0.0002 | Phosphatidylinositol transfer protein (PITP) controlled by the multiple drug resistance regulator Pdr1p, localizes to lipid particles and microsomes, controls levels of various lipids, may regulate lipid synthesis, homologous to Pdr17p |
| YLR142W | PUT1 | -2.39 | 7.11e-05 | Proline oxidase, nuclear-encoded mitochondrial protein involved in utilization of proline as sole nitrogen source; PUT1 transcription is induced by Put3p in the presence of proline and the absence of a preferred nitrogen source |
| YOR348C | PUT4 | -2.36 | 8.64e-08 | Proline permease, required for high-affinity transport of proline; also transports the toxic proline analog azetidine-2-carboxylate (AzC); PUT4 transcription is repressed in ammonia-grown cells |
| YHR029C | YHI9 | -2.36 | 6.32e-08 | Protein of unknown function; null mutant is defective in unfolded protein response; possibly involved in a membrane regulation metabolic pathway; member of the PhzF superfamily, though most likely not involved in phenazine production |
| YNL112W | DBP2 | -2.28 | 0.0002 | Essential ATP-dependent RNA helicase of the DEAD-box protein family, involved in nonsense-mediated mRNA decay and rRNA processing |
| YKR034W | DAL80 | -2.15 | 1.44e-06 | Negative regulator of genes in multiple nitrogen degradation pathways; expression is regulated by nitrogen levels and by Gln3p; member of the GATA-binding family, forms homodimers and heterodimers with Deh1p |
| YER049W | TPA1 | -2.11 | 7.15e-05 | Protein of unknown function; interacts with Sup45p (eRF1), Sup35p (eRF3) and Pab1p; has a role in translation termination efficiency, mRNA poly(A) tail length and mRNA stability |
| YMR088C | VBA1 | -2.08 | 6.94e-05 | Permease of basic amino acids in the vacuolar membrane |
| YNL111C | CYB5 | -2.08 | 0.0002 | Cytochrome b5, involved in the sterol and lipid biosynthesis pathways; acts as an electron donor to support sterol C5-6 desaturation |
| YGR035C | . | -2.06 | 9.08e-05 | Putative protein of unknown function, potential Cdc28p substrate; transcription is activated by paralogous transcription factors Yrm1p and Yrr1p along with genes involved in multidrug resistance |
| YMR220W | ERG8 | -2.05 | 9.17e-06 | Phosphomevalonate kinase, an essential cytosolic enzyme that acts in the biosynthesis of isoprenoids and sterols, including ergosterol, from mevalonate |
| YGL101W | . | -1.97 | 0.0003 | Putative protein of unknown function; non-essential gene with similarity to YBR242W; interacts with the DNA helicase Hpr5p |
| YER056C | FCY2 | -1.96 | 3.39e-05 | Purine-cytosine permease, mediates purine (adenine, guanine, and hypoxanthine) and cytosine accumulation |
| YJR152W | DAL5 | -1.95 | 0.0013 | Allantoin permease; ureidosuccinate permease; expression is constitutive but sensitive to nitrogen catabolite repression |
| YDR492W | IZH1 | -1.88 | 9.38e-05 | Membrane protein involved in zinc metabolism, member of the four-protein IZH family; transcription is regulated directly by Zap1p, expression induced by zinc deficiency and fatty acids; deletion increases sensitivity to elevated zinc |
| YCL036W | GFD2 | -1.87 | 0.0014 | Protein of unknown function, identified as a high-copy suppressor of a dbp5 mutation |
| YPR061C | JID1 | -1.86 | 3.87e-05 | Probable Hsp40p co-chaperone, has a DnaJ-like domain and appears to be involved in ER-associated degradation of misfolded proteins containing a tightly folded cytoplasmic domain; inhibits replication of Brome mosaic virus in S. cerevisiae |
| YEL034W | HYP2 | -1.85 | 0.0006 | Translation initiation factor eIF-5A, promotes formation of the first peptide bond; similar to and functionally redundant with Anb1p; possible role in translation elongation; undergoes an essential hypusination modification |
| YNL141W | AAH1 | -1.79 | 7.65e-05 | Adenine deaminase (adenine aminohydrolase), converts adenine to hypoxanthine; involved in purine salvage; transcriptionally regulated by nutrient levels and growth phase; Aah1p degraded upon entry into quiescence via SCF and the proteasome |
| YBL095W | . | -1.79 | 8.77e-05 | Putative protein of unknown function; the authentic, non-tagged protein is detected in highly purified mitochondria in high-throughput studies |
| YHR128W | FUR1 | -1.75 | 1.38e-05 | Uracil phosphoribosyltransferase, synthesizes UMP from uracil; involved in the pyrimidine salvage pathway |
| YMR070W | MOT3 | -1.74 | 9.17e-05 | Nuclear transcription factor with two Cys2-His2 zinc fingers; involved in repression of a subset of hypoxic genes by Rox1p, repression of several DAN/TIR genes during aerobic growth, and repression of ergosterol biosynthetic genes |
| YBR242W | . | -1.72 | 9.74e-05 | Putative protein of unknown function; green fluorescent protein (GFP)-fusion protein localizes to the cytoplasm and nucleus; YBR242W is not an essential gene |
| YPL028W | ERG10 | -1.61 | 0.0001 | Acetyl-CoA C-acetyltransferase (acetoacetyl-CoA thiolase), cytosolic enzyme that transfers an acetyl group from one acetyl-CoA molecule to another, forming acetoacetyl-CoA; involved in the first step in mevalonate biosynthesis |
| YHR048W | YHK8 | -1.59 | 0.0013 | Presumed antiporter of the DHA1 family of multidrug resistance transporters; contains 12 predicted transmembrane spans; expression of gene is up-regulated in cells exhibiting reduced susceptibility to azoles |
| YNR019W | ARE2 | -1.59 | 4.40e-05 | Acyl-CoA:sterol acyltransferase, isozyme of Are1p; endoplasmic reticulum enzyme that contributes the major sterol esterification activity in the presence of oxygen |
| YKR039W | GAP1 | -1.58 | 0.0007 | General amino acid permease; localization to the plasma membrane is regulated by nitrogen source |
| YLL012W | YEH1 | -1.56 | 0.0004 | Steryl ester hydrolase, one of three gene products (Yeh1p, Yeh2p, Tgl1p) responsible for steryl ester hydrolase activity and involved in sterol homeostasis; localized to lipid particle membranes |
| YGR159C | NSR1 | -1.55 | 0.0004 | Nucleolar protein that binds nuclear localization sequences, required for pre-rRNA processing and ribosome biogenesis |
| YJR048W | CYC1 | -1.53 | 0.0006 | Cytochrome c, isoform 1; electron carrier of the mitochondrial intermembrane space that transfers electrons from ubiquinone-cytochrome c oxidoreductase to cytochrome c oxidase during cellular respiration |
| YDR037W | KRS1 | -1.53 | 0.0006 | Lysyl-tRNA synthetase |
| YPR065W | ROX1 | -1.51 | 0.0006 | Heme-dependent repressor of hypoxic genes; contains an HMG domain that is responsible for DNA bending activity |
| YGR125W | . | -1.50 | 3.47e-06 | Putative protein of unknown function; deletion mutant has decreased rapamycin resistance but normal wormannin resistance; green fluorescent protein (GFP)-fusion protein localizes to the vacuole |
| YFR055W | IRC7 | -1.50 | 6.00e-06 | Putative cystathionine beta-lyase; involved in copper ion homeostasis and sulfur metabolism; null mutant displays increased levels of spontaneous Rad52p foci; expression induced by nitrogen limitation in a GLN3, GAT1-dependent manner |
| YML126C | ERG13 | -1.49 | 0.0004 | 3-hydroxy-3-methylglutaryl-CoA (HMG-CoA) synthase, catalyzes the formation of HMG-CoA from acetyl-CoA and acetoacetyl-CoA; involved in the second step in mevalonate biosynthesis |
| YBL043W | ECM13 | -1.48 | 0.0023 | Non-essential protein of unknown function; induced by treatment with 8-methoxypsoralen and UVA irradiation |
| YPL117C | IDI1 | -1.47 | 0.0002 | Isopentenyl diphosphate:dimethylallyl diphosphate isomerase (IPP isomerase), catalyzes an essential activation step in the isoprenoid biosynthetic pathway; required for viability |
| YER145C | FTR1 | -1.47 | 0.0002 | High affinity iron permease involved in the transport of iron across the plasma membrane; forms complex with Fet3p; expression is regulated by iron |
| YLR153C | ACS2 | -1.44 | 0.0009 | Acetyl-coA synthetase isoform which, along with Acs1p, is the nuclear source of acetyl-coA for histone acetylation; mutants affect global transcription; required for growth on glucose; expressed under anaerobic conditions |
| YGR175C | ERG1 | -1.41 | 7.75e-05 | Squalene epoxidase, catalyzes the epoxidation of squalene to 2,3-oxidosqualene; plays an essential role in the ergosterol-biosynthesis pathway and is the specific target of the antifungal drug terbinafine |
| YFL051C | . | -1.39 | 2.16e-06 | Putative protein of unknown function; YFL051C is not an essential gene |
| YMR318C | ADH6 | -1.38 | 0.0008 | NADPH-dependent medium chain alcohol dehydrogenase with broad substrate specificity; member of the cinnamyl family of alcohol dehydrogenases; may be involved in fusel alcohol synthesis or in aldehyde tolerance |
| YJL196C | ELO1 | -1.38 | 0.0001 | Elongase I, medium-chain acyl elongase, catalyzes carboxy-terminal elongation of unsaturated C12-C16 fatty acyl-CoAs to C16-C18 fatty acids |
| YPR194C | OPT2 | -1.36 | 0.0016 | Oligopeptide transporter; member of the OPT family, with potential orthologs in S. pombe and C. albicans |
| YKR071C | DRE2 | -1.36 | 0.0016 | Protein of unknown function required for iron-sulfur cluster assembly and sister chromatid cohesion; mutation displays synthetic lethal interaction with the pol3-13 allele of CDC2 |
| YHR039C | MSC7 | -1.36 | 0.0001 | Protein of unknown function, green fluorescent protein (GFP)-fusion protein localizes to the endoplasmic reticulum; msc7 mutants are defective in directing meiotic recombination events to homologous chromatids |
| YGR128C | UTP8 | -1.35 | 0.0042 | Nucleolar protein required for export of tRNAs from the nucleus; also copurifies with the small subunit (SSU) processome containing the U3 snoRNA that is involved in processing of pre-18S rRNA |
| YOL128C | YGK3 | -1.35 | 1.60e-05 | Protein kinase related to mammalian glycogen synthase kinases of the GSK-3 family; GSK-3 homologs (Mck1p, Rim11p, Mrk1p, Ygk3p) are involved in control of Msn2p-dependent transcription of stress responsive genes and in protein degradation |
| YMR217W | GUA1 | -1.34 | 0.0034 | GMP synthase, an enzyme that catalyzes the second step in the biosynthesis of GMP from inosine 5-phosphate (IMP); transcription is not subject to regulation by guanine but is negatively regulated by nutrient starvation |
| YEL066W | HPA3 | -1.32 | 1.84e-06 | D-Amino acid N-acetyltransferase, catalyzes N-acetylation of D-amino acids through ordered bi-bi mechanism in which acetyl-CoA is first substrate bound and CoA is last product liberated; similar to Hpa2p, acetylates histones weakly in vitro |
| YPL265W | DIP5 | -1.30 | 1.76e-05 | Dicarboxylic amino acid permease, mediates high-affinity and high-capacity transport of L-glutamate and L-aspartate; also a transporter for Gln, Asn, Ser, Ala, and Gly |
| YOR051C | . | -1.30 | 0.0058 | Nuclear protein that inhibits replication of Brome mosaic virus in S. cerevisiae, which is a model system for studying replication of positive-strand RNA viruses in their natural hosts |
| YHR007C | ERG11 | -1.29 | 0.0023 | Lanosterol 14-alpha-demethylase, catalyzes the C-14 demethylation of lanosterol to form 4,4-dimethyl cholesta-8,14,24-triene-3-beta-ol in the ergosterol biosynthesis pathway; member of the cytochrome P450 family |
| YKL056C | TMA19 | -1.27 | 0.0081 | Protein that associates with ribosomes; homolog of translationally controlled tumor protein; green fluorescent protein (GFP)-fusion protein localizes to the cytoplasm and relocates to the mitochondrial outer surface upon oxidative stress |
| YDR165W | TRM82 | -1.25 | 0.0002 | Subunit of a tRNA methyltransferase complex composed of Trm8p and Trm82p that catalyzes 7-methylguanosine modification of tRNA |
| YML008C | ERG6 | -1.25 | 0.0008 | Delta(24)-sterol C-methyltransferase, converts zymosterol to fecosterol in the ergosterol biosynthetic pathway by methylating position C-24; localized to both lipid particles and mitochondrial outer membrane |
| YNR053C | NOG2 | -1.24 | 0.0036 | Putative GTPase that associates with pre-60S ribosomal subunits in the nucleolus and is required for their nuclear export and maturation |
| YOL124C | TRM11 | -1.23 | 0.0012 | Catalytic subunit of an adoMet-dependent tRNA methyltransferase complex (Trm11p-Trm112p), required for the methylation of the guanosine nucleotide at position 10 (m2G10) in tRNAs; contains a THUMP domain and a methyltransferase domain |
| YGL077C | HNM1 | -1.23 | 2.40e-06 | Choline/ethanolamine transporter; involved in the uptake of nitrogen mustard and the uptake of glycine betaine during hypersaline stress; co-regulated with phospholipid biosynthetic genes and negatively regulated by choline and myo-inositol |
| YBR213W | MET8 | -1.22 | 0.0027 | Bifunctional dehydrogenase and ferrochelatase, involved in the biosynthesis of siroheme, a prosthetic group used by sulfite reductase; required for sulfate assimilation and methionine biosynthesis |
| YMR015C | ERG5 | -1.22 | 5.59e-05 | C-22 sterol desaturase, a cytochrome P450 enzyme that catalyzes the formation of the C-22(23) double bond in the sterol side chain in ergosterol biosynthesis; may be a target of azole antifungal drugs |
| YLR168C | AIM30 | -1.22 | 0.0023 | Putative protein of unknown function that may be involved in intramitochondrial sorting; similar to Ups1p and to human PRELI; GFP-tagged protein localizes to mitochondria; required for wild-type respiratory growth |
| YMR202W | ERG2 | -1.22 | 2.41e-05 | C-8 sterol isomerase, catalyzes the isomerization of the delta-8 double bond to the delta-7 position at an intermediate step in ergosterol biosynthesis |
| YHR094C | HXT1 | -1.21 | 5.01e-07 | Low-affinity glucose transporter of the major facilitator superfamily, expression is induced by Hxk2p in the presence of glucose and repressed by Rgt1p when glucose is limiting |
| YLR205C | HMX1 | -1.21 | 0.0024 | ER localized, heme-binding peroxidase involved in the degradation of heme; does not exhibit heme oxygenase activity despite similarity to heme oxygenases; expression regulated by AFT1 |
| YPL245W | . | -1.19 | 1.43e-05 | Putative protein of unknown function; green fluorescent protein (GFP)-fusion protein localizes to both the nucleus and the cytoplasm |
| YEL026W | SNU13 | -1.18 | 0.000156919581997246 | RNA binding protein, part of U3 snoRNP involved in rRNA processing, part of U4/U6-U5 tri-snRNP involved in mRNA splicing, similar to human 15.5K protein |
| YML075C | HMG1 | -1.17 | 0.0063 | One of two isozymes of HMG-CoA reductase that catalyzes the conversion of HMG-CoA to mevalonate, which is a rate-limiting step in sterol biosynthesis; localizes to the nuclear envelope; overproduction induces the formation of karmellae |
| YJL025W | RRN7 | -1.16 | 0.0014 | Protein involved in the transcription of 35S rRNA genes by RNA polymerase I; component of the core factor (CF) complex also composed of Rrn11p, Rrn6p and TATA-binding protein |
| YLR197W | NOP56 | -1.16 | 0.0006 | Essential evolutionarily-conserved nucleolar protein component of the box C/D snoRNP complexes that direct 2-O-methylation of pre-rRNA during its maturation; overexpression causes spindle orientation defects |
| YOL058W | ARG1 | -1.16 | 6.28e-06 | Arginosuccinate synthetase, catalyzes the formation of L-argininosuccinate from citrulline and L-aspartate in the arginine biosynthesis pathway; potential Cdc28p substrate |
| YGR052W | FMP48 | -1.15 | 0.0010 | Putative protein of unknown function; the authentic, non-tagged protein is detected in highly purified mitochondria in high-throughput studies; induced by treatment with 8-methoxypsoralen and UVA irradiation |
| YNL129W | NRK1 | -1.15 | 4.30e-05 | Nicotinamide riboside kinase, catalyzes the phosphorylation of nicotinamide riboside and nicotinic acid riboside in salvage pathways for NAD+ biosynthesis |
| YLR126C | . | -1.14 | 7.71e-05 | Putative protein of unknown function with similarity to glutamine amidotransferase proteins; has Aft1p-binding motif in the promoter; may be involved in copper and iron homeostasis; YLR126C is not an essential protein |
| YDL236W | PHO13 | -1.14 | 0.0001 | Alkaline phosphatase specific for p-nitrophenyl phosphate, involved in dephosphorylation of histone II-A and casein |
| YOR095C | RKI1 | -1.13314445638921 | 0.0014 | Ribose-5-phosphate ketol-isomerase, catalyzes the interconversion of ribose 5-phosphate and ribulose 5-phosphate in the pentose phosphate pathway; participates in pyridoxine biosynthesis |
| YMR208W | ERG12 | -1.13 | 0.0046 | Mevalonate kinase, acts in the biosynthesis of isoprenoids and sterols, including ergosterol, from mevalonate |
| YDR299W | BFR2 | -1.12 | 0.0001 | Essential protein possibly involved in secretion; multicopy suppressor of sensitivity to Brefeldin A |
| YLR432W | IMD3 | -1.12 | 0.0004 | Inosine monophosphate dehydrogenase, catalyzes the first step of GMP biosynthesis, member of a four-gene family in S. cerevisiae, constitutively expressed |
| YER131W | RPS26B | -1.12 | 0.0050 | Protein component of the small (40S) ribosomal subunit; nearly identical to Rps26Ap and has similarity to rat S26 ribosomal protein |
| YOR108W | LEU9 | -1.12 | 0.0034 | Alpha-isopropylmalate synthase II (2-isopropylmalate synthase), catalyzes the first step in the leucine biosynthesis pathway; the minor isozyme, responsible for the residual alpha-IPMS activity detected in a leu4 null mutant |
| YML043C | RRN11 | -1.12 | 0.0011 | Protein required for rDNA transcription by RNA polymerase I, component of the core factor (CF) of rDNA transcription factor, which also contains Rrn6p and Rrn7p |
| YCL054W | SPB1 | -1.11 | 7.74e-05 | AdoMet-dependent methyltransferase involved in rRNA processing and 60S ribosomal subunit maturation; methylates G2922 in the tRNA docking site of the large subunit rRNA and in the absence of snR52, U2921; suppressor of PAB1 mutants |
| YNR043W | MVD1 | -1.11 | 0.0001 | Mevalonate pyrophosphate decarboxylase, essential enzyme involved in the biosynthesis of isoprenoids and sterols, including ergosterol; acts as a homodimer |
| YGL224C | SDT1 | -1.10 | 1.15e-05 | Pyrimidine nucleotidase; overexpression suppresses the 6-AU sensitivity of transcription elongation factor S-II, as well as resistance to other pyrimidine derivatives |
| YGR177C | ATF2 | -1.09 | 8.32e-05 | Alcohol acetyltransferase, may play a role in steroid detoxification; forms volatile esters during fermentation, which is important in brewing |
| YKL029C | MAE1 | -1.08 | 0.0038 | Mitochondrial malic enzyme, catalyzes the oxidative decarboxylation of malate to pyruvate, which is a key intermediate in sugar metabolism and a precursor for synthesis of several amino acids |
| YMR131C | RRB1 | -1.08693367364473 | 0.0009 | Essential nuclear protein involved in early steps of ribosome biogenesis; physically interacts with the ribosomal protein Rpl3p |
| YNL175C | NOP13 | -1.08 | 6.70e-05 | Protein of unknown function, localizes to the nucleolus and nucleoplasm; contains an RNA recognition motif (RRM) and has similarity to Nop12p, which is required for processing of pre-18S rRNA |
| YBR104W | YMC2 | -1.07 | 0.0009 | Mitochondrial protein, putative inner membrane transporter with a role in oleate metabolism and glutamate biosynthesis; member of the mitochondrial carrier (MCF) family; has similarity with Ymc1p |
| YGR234W | YHB1 | -1.06 | 0.0042 | Nitric oxide oxidoreductase, flavohemoglobin involved in nitric oxide detoxification; plays a role in the oxidative and nitrosative stress responses |
| YER060W-A | FCY22 | -1.06422069887205 | 5.12e-06 | Putative purine-cytosine permease, very similar to Fcy2p but cannot substitute for its function |
| YMR134W | ERG29 | -1.06 | 0.0002 | Protein that binds to and regulates Erg25p; localized to the ER; null mutant is viable in a respiratory defective background; synthetic lethal with mmt1 and mmt2 mutations; highly conserved in ascomycetes |
| YDR465C | RMT2 | -1.05 | 0.0021 | Arginine methyltransferase; ribosomal protein L12 is a substrate |
| YDR090C | . | -1.04 | 0.0002 | Putative protein of unknown function |
| YNR012W | URK1 | -1.04 | 1.44e-06 | Uridine/cytidine kinase, component of the pyrimidine ribonucleotide salvage pathway that converts uridine into UMP and cytidine into CMP; involved in the pyrimidine deoxyribonucleotide salvage pathway, converting deoxycytidine into dCMP |
| YLR056W | ERG3 | -1.03 | 0.0001 | C-5 sterol desaturase, catalyzes the introduction of a C-5(6) double bond into episterol, a precursor in ergosterol biosynthesis; mutants are viable, but cannot grow on non-fermentable carbon sources |
| YLR108C | . | -1.03 | 0.0001 | Protein of unknown function; green fluorescent protein (GFP)-fusion protein localizes to the nucleus; YLR108C is not an esssential gene |
| YJL167W | ERG20 | -1.03 | 1.44e-05 | Farnesyl pyrophosphate synthetase, has both dimethylallyltranstransferase and geranyltranstransferase activities; catalyzes the formation of C15 farnesyl pyrophosphate units for isoprenoid and sterol biosynthesis |
| YDR060W | MAK21 | -1.02 | 0.0007 | Constituent of 66S pre-ribosomal particles, required for large (60S) ribosomal subunit biogenesis; involved in nuclear export of pre-ribosomes; required for maintenance of dsRNA virus; homolog of human CAATT-binding protein |
| YKR060W | UTP30 | -1.01 | 7.46e-06 | Subunit of U3-containing 90S preribosome complex involved in production of 18S rRNA and assembly of small ribosomal subunit |
| YPL107W | . | -1.00 | 0.0005 | Putative protein of unknown function; green fluorescent protein (GFP)-fusion protein localizes to mitochondria; YPL107W is not an essential gene |
|  |  |  |  |  |
