# Supplementary material for: Impact of Nutrient Imbalance on Wine Alcoholic Fermentations: Nitrogen Excess Enhances Yeast Cell Death in Lipid-Limited Must
Source: PLoS One. 2013 Apr 26;8(4):e61645. doi: 10.1371/journal.pone.0061645 (PMC3637302; doi:10.1371/journal.pone.0061645)
Supplement: Table S3 — Genes that were significantly up-regulated in the 59A SCH9 -deleted mutant. (DOC) [file pone.0061645.s008.doc]

**Table S3 Genes that were significantly up-regulated in the 59A *SCH9*-deleted mutant.**

| Systematic name | Common name | logFC | P value | SGD Annotation |
| --- | --- | --- | --- | --- |
| YDR216W | ADR1 | 1.01 | 1.50e-06 | Carbon source-responsive zinc-finger transcription factor, required for transcription of the glucose-repressed gene ADH2, of peroxisomal protein genes, and of genes required for ethanol, glycerol, and fatty acid utilization |
| YPR005C | HAL1 | 1.01 | 2.55e-06 | Cytoplasmic protein involved in halotolerance; decreases intracellular Na+ (via Ena1p) and increases intracellular K+ by decreasing efflux; expression repressed by Ssn6p-Tup1p and Sko1p and induced by NaCl, KCl, and sorbitol through Gcn4p |
| YPR149W | NCE102 | 1.01 | 0.0023 | Protein of unknown function; contains transmembrane domains; involved in secretion of proteins that lack classical secretory signal sequences; component of the detergent-insoluble glycolipid-enriched complexes (DIGs) |
| YNL144C | . | 1.03 | 0.0001 | Putative protein of unknown function; the authentic, non-tagged protein is detected in highly purified mitochondria in high-throughput studies; YNL144C is not an essential gene |
| YML047C | PRM6 | 1.03 | 5.44e-06 | Pheromone-regulated protein, predicted to have 2 transmembrane segments; regulated by Ste12p during mating |
| YNL058C | . | 1.03 | 1.26e-05 | Putative protein of unknown function; green fluorescent protein (GFP)-fusion protein localizes to the vacuole; YNL058C is not an essential gene |
| YLL026W | HSP104 | 1.03 | 0.0004 | Heat shock protein that cooperates with Ydj1p (Hsp40) and Ssa1p (Hsp70) to refold and reactivate previously denatured, aggregated proteins; responsive to stresses including: heat, ethanol, and sodium arsenite; involved in [PSI+] propagation |
| YNL241C | ZWF1 | 1.039 | 0.0037 | Glucose-6-phosphate dehydrogenase (G6PD), catalyzes the first step of the pentose phosphate pathway; involved in adapting to oxidatve stress; homolog of the human G6PD which is deficient in patients with hemolytic anemia |
| YEL070W | DSF1 | 1.04 | 4.96e-06 | Deletion suppressor of mpt5 mutation |
| YIR014W | . | 1.04 | 1.46e-05 | Putative protein of unknown function; green fluorescent protein (GFP)-fusion protein localizes to the vacuole; expression directly regulated by the metabolic and meiotic transcriptional regulator Ume6p; YIR014W is a non-essential gene |
| YDL197C | ASF2 | 1.04 | 0.0033 | Anti-silencing protein that causes derepression of silent loci when overexpressed |
| YGL248W | PDE1 | 1.05 | 0.0023 | Low-affinity cyclic AMP phosphodiesterase, controls glucose and intracellular acidification-induced cAMP signaling, target of the cAMP-protein kinase A (PKA) pathway; glucose induces transcription and inhibits translation |
| YAL054C | ACS1 | 1.05 | 1.88e-06 | Acetyl-coA synthetase isoform which, along with Acs2p, is the nuclear source of acetyl-coA for histone acetlyation; expressed during growth on nonfermentable carbon sources and under aerobic conditions |
| YHR137W | ARO9 | 1.05 | 2.07e-06 | Aromatic aminotransferase II, catalyzes the first step of tryptophan, phenylalanine, and tyrosine catabolism |
| YAL060W | BDH1 | 1.05 | 3.43e-06 | NAD-dependent (R,R)-butanediol dehydrogenase, catalyzes oxidation of (R,R)-2,3-butanediol to (3R)-acetoin, oxidation of meso-butanediol to (3S)-acetoin, and reduction of acetoin; enhances use of 2,3-butanediol as an aerobic carbon source |
| YCR091W | KIN82 | 1.06 | 0.0002 | Putative serine/threonine protein kinase, most similar to cyclic nucleotide-dependent protein kinase subfamily and the protein kinase C subfamily |
| YER150W | SPI1 | 1.07 | 9.20e-06 | GPI-anchored cell wall protein involved in weak acid resistance; basal expression requires Msn2p/Msn4p; expression is induced under conditions of stress and during the diauxic shift; similar to Sed1p |
| YLR438W | CAR2 | 1.07 | 0.0006 | L-ornithine transaminase (OTAse), catalyzes the second step of arginine degradation, expression is dually-regulated by allophanate induction and a specific arginine induction process; not nitrogen catabolite repression sensitive |
| YJL045W | . | 1.07 | 1.95e-05 | Minor succinate dehydrogenase isozyme; homologous to Sdh1p, the major isozyme reponsible for the oxidation of succinate and transfer of electrons to ubiquinone; induced during the diauxic shift in a Cat8p-dependent manner |
| YBR018C | GAL7 | 1.07 | 7.64e-06 | Galactose-1-phosphate uridyl transferase, synthesizes glucose-1-phosphate and UDP-galactose from UDP-D-glucose and alpha-D-galactose-1-phosphate in the second step of galactose catabolism |
| YOL011W | PLB3 | 1.08 | 9.72e-06 | Phospholipase B (lysophospholipase) involved in phospholipid metabolism; hydrolyzes phosphatidylinositol and phosphatidylserine and displays transacylase activity in vitro |
| YKR009C | FOX2 | 1.09 | 4.27e-05 | Multifunctional enzyme of the peroxisomal fatty acid beta-oxidation pathway; has 3-hydroxyacyl-CoA dehydrogenase and enoyl-CoA hydratase activities |
| YFL027C | GYP8 | 1.10 | 1.23e-06 | GTPase-activating protein for yeast Rab family members; Ypt1p is the preferred in vitro substrate but also acts on Sec4p, Ypt31p and Ypt32p; involved in the regulation of ER to Golgi vesicle transport |
| YHR206W | SKN7 | 1.10 | 4.41e-06 | Nuclear response regulator and transcription factor, part of a branched two-component signaling system; required for optimal induction of heat-shock genes in response to oxidative stress; involved in osmoregulation |
| YPR177C | . | 1.10 | 6.82e-06 | Dubious open reading frame unlikely to encode a protein, based on available experimental and comparative sequence data; partially overlaps the 5 end of the essential PRP4 gene encoding a component of the U4/U6-U5 snRNP complex |
| YKL050C | . | 1.11 | 0.0002 | Protein of unknown function; the YKL050W protein is a target of the SCFCdc4 ubiquitin ligase complex and YKL050W transcription is regulated by Azf1p |
| YKL085W | MDH1 | 1.11 | 0.0002 | Mitochondrial malate dehydrogenase, catalyzes interconversion of malate and oxaloacetate; involved in the tricarboxylic acid (TCA) cycle; phosphorylated |
| YPL111W | CAR1 | 1.12 | 9.13e-07 | Arginase, responsible for arginine degradation, expression responds to both induction by arginine and nitrogen catabolite repression; disruption enhances freeze tolerance |
| YOR086C | TCB1 | 1.12 | 1.92e-05 | Lipid-binding protein containing three calcium and lipid binding domains; non-tagged protein localizes to mitochondria and GFP-fusion protein localizes to the cell periphery; C-termini of Tcb1p, Tcb2p and Tcb3p interact |
| YLR121C | YPS3 | 1.12 | 2.60e-06 | Aspartic protease, attached to the plasma membrane via a glycosylphosphatidylinositol (GPI) anchor |
| YNL093W | YPT53 | 1.14 | 0.0011 | GTPase, similar to Ypt51p and Ypt52p and to mammalian rab5; required for vacuolar protein sorting and endocytosis |
| YGL089C | MF(ALPHA)2 | 1.15 | 6.43e-05 | Mating pheromone alpha-factor, made by alpha cells; interacts with mating type a cells to induce cell cycle arrest and other responses leading to mating; also encoded by MF(ALPHA)1, which is more highly expressed than MF(ALPHA)2 |
| YOR161C | PNS1 | 1.15 | 0.0002 | Protein of unknown function; has similarity to Torpedo californica tCTL1p, which is postulated to be a choline transporter, neither null mutation nor overexpression affects choline transport |
| YMR104C | YPK2 | 1.15 | 0.0003 | Protein kinase with similarityto serine/threonine protein kinase Ypk1p; functionally redundant with YPK1 at the genetic level; participates in a signaling pathway required for optimal cell wall integrity; homolog of mammalian kinase SGK |
| YIR019C | MUC1 | 1.16 | 8.04e-06 | GPI-anchored cell surface glycoprotein (flocculin) required for pseudohyphal formation, invasive growth, flocculation, and biofilms; transcriptionally regulated by the MAPK pathway (via Ste12p and Tec1p) and the cAMP pathway (via Flo8p) |
| YKR076W | ECM4 | 1.17 | 2.29e-07 | Omega class glutathione transferase; not essential; similar to Ygr154cp; green fluorescent protein (GFP)-fusion protein localizes to the cytoplasm |
| YBL016W | FUS3 | 1.17 | 2.24e-05 | Mitogen-activated serine/threonine protein kinase involved in mating; phosphoactivated by Ste7p; substrates include Ste12p, Far1p, Bni1p, Sst2p; inhibits invasive growth during mating by phosphorylating Tec1p, promoting its degradation |
| YKL026C | GPX1 | 1.17 | 0.0002 | Phospholipid hydroperoxide glutathione peroxidase induced by glucose starvation that protects cells from phospholipid hydroperoxides and nonphospholipid peroxides during oxidative stress |
| YKL163W | PIR3 | 1.17 | 4.74e-06 | O-glycosylated covalently-bound cell wall protein required for cell wall stability; expression is cell cycle regulated, peaking in M/G1 and also subject to regulation by the cell integrity pathway |
| YIL136W | OM45 | 1.18 | 1.21e-06 | Protein of unknown function, major constituent of the mitochondrial outer membrane; located on the outer (cytosolic) face of the outer membrane |
| YDR046C | BAP3 | 1.19 | 5.24e-07 | Amino acid permease involved in the uptake of cysteine, leucine, isoleucine and valine |
| YBR067C | TIP1 | 1.20 | 1.12e-06 | Major cell wall mannoprotein with possible lipase activity; transcription is induced by heat- and cold-shock; member of the Srp1p/Tip1p family of serine-alanine-rich proteins |
| YMR101C | SRT1 | 1.21 | 2.16e-05 | Cis-prenyltransferase involved in synthesis of long-chain dolichols (19-22 isoprene units; as opposed to Rer2p which synthesizes shorter-chain dolichols); localizes to lipid bodies; transcription is induced during stationary phase |
| YNL042W | BOP3 | 1.21 | 3.53e-06 | Protein of unknown function, potential Cdc28p substrate; overproduction suppresses a pam1 slv3 double null mutation and confers resistance to methylmercury |
| YMR206W | . | 1.21 | 7.19e-06 | Putative protein of unknown function; YMR206W is not an essential gene |
| YNL279W | PRM1 | 1.21 | 0.0023 | Pheromone-regulated multispanning membrane protein involved in membrane fusion during mating; predicted to have 5 transmembrane segments and a coiled coil domain; localizes to the shmoo tip; regulated by Ste12p |
| YLR398C | SKI2 | 1.22 | 2.08e-05 | Putative RNA helicase, involved in exosome mediated 3 to 5 mRNA degradation and translation inhibition of non-poly(A) mRNAs; forms complex with Ski3p and Ski8p; required for repressing propagation of dsRNA viruses |
| YPR138C | MEP3 | 1.22 | 1.71e-06 | Ammonium permease of high capacity and low affinity; belongs to a ubiquitous family of cytoplasmic membrane proteins that transport only ammonium (NH4+); expression is under the nitrogen catabolite repression regulation ammonia permease |
| YMR065W | KAR5 | 1.22 | 0.0001 | Protein required for nuclear membrane fusion during karyogamy, localizes to the membrane with a soluble portion in the endoplasmic reticulum lumen, may form a complex with Jem1p and Kar2p; expression of the gene is regulated by pheromone |
| YDR277C | MTH1 | 1.23 | 3.23e-05 | Negative regulator of the glucose-sensing signal transduction pathway, required for repression of transcription by Rgt1p; interacts with Rgt1p and the Snf3p and Rgt2p glucose sensors; phosphorylated by Yck1p, triggering Mth1p degradation |
| YKL086W | SRX1 | 1.24 | 6.83e-05 | Sulfiredoxin, contributes to oxidative stress resistance by reducing cysteine-sulfinic acid groups in the peroxiredoxins Tsa1p and Ahp1p that are formed upon exposure to oxidants; conserved in higher eukaryotes |
| YLR304C | ACO1 | 1.26 | 6.64e-06 | Aconitase, required for the tricarboxylic acid (TCA) cycle and also independently required for mitochondrial genome maintenance; phosphorylated; component of the mitochondrial nucleoid; mutation leads to glutamate auxotrophy |
| YDL085W | NDE2 | 1.27 | 1.22e-05 | Mitochondrial external NADH dehydrogenase, catalyzes the oxidation of cytosolic NADH; Nde1p and Nde2p are involved in providing the cytosolic NADH to the mitochondrial respiratory chain |
| YOR186W | . | 1.28 | 5.66e-06 | Putative protein of unknown function; proper regulation of expression during heat stress is sphingolipid-dependent |
| YIL101C | XBP1 | 1.28 | 6.33e-06 | Transcriptional repressor that binds to promoter sequences of the cyclin genes, CYS3, and SMF2; expression is induced by stress or starvation during mitosis, and late in meiosis; member of the Swi4p/Mbp1p family; potential Cdc28p substrate |
| YPR030W | CSR2 | 1.29 | 0.0004 | Nuclear protein with a potential regulatory role in utilization of galactose and nonfermentable carbon sources; overproduction suppresses the lethality at high temperature of a chs5 spa2 double null mutation; potential Cdc28p substrate |
| YLR213C | CRR1 | 1.30 | 5.48e-05 | Putative glycoside hydrolase of the spore wall envelope; required for normal spore wall assembly, possibly for cross-linking between the glucan and chitosan layers; expressed during sporulation |
| YOR248W | . | 1.31 | 3.93e-06 | Dubious open reading frame unlikely to encode a functional protein, based on available experimental and comparative sequence data |
| YKL107W | . | 1.31 | 0.0001 | Putative protein of unknown function |
| YHR097C | . | 1.32 | 1.04e-06 | Putative protein of unknown function; green fluorescent protein (GFP)-fusion protein localizes to the cytoplasm and the nucleus |
| YNL033W | . | 1.33 | 3.40e-06 | Putative protein of unknown function |
| YCL055W | KAR4 | 1.34 | 1.05e-06 | Transcription factor required for gene regulation in repsonse to pheromones; also required during meiosis; exists in two forms, a slower-migrating form more abundant during vegetative growth and a faster-migrating form induced by pheromone |
| YER124C | DSE1 | 1.34 | 6.61e-07 | Daughter cell-specific protein, may participate in pathways regulating cell wall metabolism; deletion affects cell separation after division and sensitivity to drugs targeted against the cell wall |
| YNL145W | MFA2 | 1.39 | 8.45e-06 | Mating pheromone a-factor, made by a cells; interacts with alpha cells to induce cell cycle arrest and other responses leading to mating; biogenesis involves C-terminal modification, N-terminal proteolysis, and export; also encoded by MFA1 |
| YPL004C | LSP1 | 1.41 | 5.22e-05 | Primary component of eisosomes, which are large immobile patch structures at the cell cortex associated with endocytosis, along with Pil1p and Sur7p; null mutants show activation of Pkc1p/Ypk1p stress resistance pathways |
| YGR066C | . | 1.41 | 0.0032 | Putative protein of unknown function |
| YDR491C | . | 1.43 | 2.55e-05 | Dubious open reading frame unlikely to encode a functional protein, based on available experimental and comparative sequence data |
| YML128C | MSC1 | 1.44 | 1.11e-06 | Protein of unknown function; mutant is defective in directing meiotic recombination events to homologous chromatids; the authentic, non-tagged protein is detected in highly purified mitochondria and is phosphorylated |
| YFL057C | AAD16 | 1.44 | 7.28e-08 | Putative aryl-alcohol dehydrogenase with similarity to P. chrysosporium aryl-alcohol dehydrogenase; mutational analysis has not yet revealed a physiological role |
| YMR107W | SPG4 | 1.45 | 2.03e-08 | Protein required for survival at high temperature during stationary phase; not required for growth on nonfermentable carbon sources |
| YJR155W | AAD10 | 1.46 | 4.40e-06 | Putative aryl-alcohol dehydrogenase with similarity to P. chrysosporium aryl-alcohol dehydrogenase; mutational analysis has not yet revealed a physiological role |
| YNL194C | . | 1.47 | 0.0002 | Integral membrane protein required for sporulation and plasma membrane sphingolipid content; has sequence similarity to SUR7 and FMP45; GFP-fusion protein is induced in response to the DNA-damaging agent MMS |
| YMR232W | FUS2 | 1.49 | 2.11e-06 | Cytoplasmic protein localized to the shmoo tip; required for the alignment of parental nuclei before nuclear fusion during mating |
| YNL019C | . | 1.49 | 4.78e-05 | Putative protein of unknown function |
| YOL052C-A | DDR2 | 1.49 | 5.56e-05 | Multistress response protein, expression is activated by a variety of xenobiotic agents and environmental or physiological stresses |
| YDR247W | VHS1 | 1.49 | 0.0001 | Cytoplasmic serine/threonine protein kinase; identified as a high-copy suppressor of the synthetic lethality of a sis2 sit4 double mutant, suggesting a role in G1/S phase progression; homolog of Sks1p |
| YMR323W | ERR3 | 1.51 | 1.80e-06 | Protein of unknown function, has similarity to enolases |
| YML007C-A | . | 1.52 | 3.76e-08 | Putative protein of unknown function; green fluorescent protein (GFP)-fusion protein localizes to mitochondria |
| YPL230W | USV1 | 1.52 | 1.91e-06 | Putative transcription factor containing a C2H2 zinc finger; mutation affects transcriptional regulation of genes involved in growth on non-fermentable carbon sources, response to salt stress and cell wall biosynthesis |
| YOR393W | ERR1 | 1.52 | 1.72e-06 | Protein of unknown function, has similarity to enolases |
| YDL182W | LYS20 | 1.53 | 2.36e-05 | Homocitrate synthase isozyme, catalyzes the condensation of acetyl-CoA and alpha-ketoglutarate to form homocitrate, which is the first step in the lysine biosynthesis pathway; highly similar to the other isozyme, Lys21p |
| YBR083W | TEC1 | 1.55 | 0.0035 | Transcription factor required for full Ty1 epxression, Ty1-mediated gene activation, and haploid invasive and diploid pseudohyphal growth; TEA/ATTS DNA-binding domain family member |
| YNR071C | . | 1.56 | 0.0008 | Putative protein of unknown function |
| YLR111W | . | 1.56 | 3.11e-06 | Dubious open reading frame unlikely to encode a protein, based on available experimental and comparative sequence data |
| YPL281C | ERR2 | 1.56 | 7.39e-07 | Protein of unknown function, has similarity to enolases |
| YIL123W | SIM1 | 1.58 | 3.28e-06 | Protein of the SUN family (Sim1p, Uth1p, Nca3p, Sun4p) that may participate in DNA replication, promoter contains SCB regulation box at -300 bp indicating that expression may be cell cycle-regulated |
| YDR461W | MFA1 | 1.59 | 1.92e-07 | Mating pheromone a-factor, made by a cells; interacts with alpha cells to induce cell cycle arrest and other responses leading to mating; biogenesis involves C-terminal modification, N-terminal proteolysis, and export; also encoded by MFA2 |
| YGR043C | NQM1 | 1.65 | 5.23e-08 | Transaldolase of unknown function; transcription is repressed by Mot1p and induced by alpha-factor and during diauxic shift; null mutant non-quiescent cells exhibit reduced reproductive capacity |
| YOR374W | ALD4 | 1.65 | 5.80e-08 | Mitochondrial aldehyde dehydrogenase, required for growth on ethanol and conversion of acetaldehyde to acetate; phosphorylated; activity is K+ dependent; utilizes NADP+ or NAD+ equally as coenzymes; expression is glucose repressed |
| YMR175W | SIP18 | 1.66 | 1.38e-05 | Protein of unknown function whose expression is induced by osmotic stress |
| YNL283C | WSC2 | 1.68 | 0.0002 | Partially redundant sensor-transducer of the stress-activated PKC1-MPK1 signaling pathway involved in maintenance of cell wall integrity and recovery from heat shock; secretory pathway Wsc2p is required for the arrest of secretion response |
| YDL204W | RTN2 | 1.68 | 8.83e-06 | Protein of unknown function; has similarity to mammalian reticulon proteins; member of the RTNLA (reticulon-like A) subfamily |
| YNL195C | . | 1.70 | 5.56e-07 | Putative protein of unknown function; shares a promoter with YNL194C; the authentic, non-tagged protein is detected in highly purified mitochondria in high-throughput studies |
| YCR089W | FIG2 | 1.72 | 1.05e-05 | Cell wall adhesin, expressed specifically during mating; may be involved in maintenance of cell wall integrity during mating |
| YGR121C | MEP1 | 1.72 | 3.32e-08 | Ammonium permease; belongs to a ubiquitous family of cytoplasmic membrane proteins that transport only ammonium (NH4+); expression is under the nitrogen catabolite repression regulation |
| YLR092W | SUL2 | 1.72 | 1.29e-07 | High affinity sulfate permease; sulfate uptake is mediated by specific sulfate transporters Sul1p and Sul2p, which control the concentration of endogenous activated sulfate intermediates |
| YOR100C | CRC1 | 1.72 | 9.80e-09 | Mitochondrial inner membrane carnitine transporter, required for carnitine-dependent transport of acetyl-CoA from peroxisomes to mitochondria during fatty acid beta-oxidation |
| YGR248W | SOL4 | 1.77 | 7.47e-06 | 6-phosphogluconolactonase with similarity to Sol3p |
| YDR380W | ARO10 | 1.88 | 1.73e-07 | Phenylpyruvate decarboxylase, catalyzes decarboxylation of phenylpyruvate to phenylacetaldehyde, which is the first specific step in the Ehrlich pathway |
| YPR127W | . | 1.90 | 1.23e-05 | Putative protein of unknown function; expression is activated by transcription factor YRM1/YOR172W; green fluorescent protein (GFP)-fusion protein localizes to both the cytoplasm and the nucleus |
| YGR088W | CTT1 | 1.91 | 1.88e-07 | Cytosolic catalase T, has a role in protection from oxidative damage by hydrogen peroxide |
| YDL222C | FMP45 | 1.92 | 2.05e-08 | Integral membrane protein localized to mitochondria (untagged protein); required for sporulation and maintaining sphingolipid content; has sequence similarity to SUR7 and YNL194C |
| YLR070C | XYL2 | 1.93 | 1.23e-07 | Xylitol dehydrogenase, converts xylitol to D-xylulose; expression induced by xylose, even though this pentose sugar is not well utilized by S. cerevisiae; null mutant has cell wall defect |
| YFL026W | STE2 | 2.02 | 0.0003 | Receptor for alpha-factor pheromone; seven transmembrane-domain GPCR that interacts with both pheromone and a heterotrimeric G protein to initiate the signaling response that leads to mating between haploid a and alpha cells |
| YBR068C | BAP2 | 2.03 | 6.99e-09 | High-affinity leucine permease, functions as a branched-chain amino acid permease involved in the uptake of leucine, isoleucine and valine; contains 12 predicted transmembrane domains |
| YGR256W | GND2 | 2.03 | 2.62e-09 | 6-phosphogluconate dehydrogenase (decarboxylating), catalyzes an NADPH regenerating reaction in the pentose phosphate pathway; required for growth on D-glucono-delta-lactone |
| YLR112W | . | 2.04 | 8.69e-06 | Dubious open reading frame unlikely to encode a protein, based on available experimental and comparative sequence data |
| YCL027W | FUS1 | 2.07 | 3.27e-08 | Membrane protein localized to the shmoo tip, required for cell fusion; expression regulated by mating pheromone; proposed to coordinate signaling, fusion, and polarization events required for fusion; potential Cdc28p substrate |
| YJL158C | CIS3 | 2.11 | 0.0007 | Mannose-containing glycoprotein constituent of the cell wall; member of the PIR (proteins with internal repeats) family |
| YDR256C | CTA1 | 2.13 | 0.0015 | Catalase A, breaks down hydrogen peroxide in the peroxisomal matrix formed by acyl-CoA oxidase (Pox1p) during fatty acid beta-oxidation |
| YBR116C | . | 2.14 | 8.51e-07 | Dubious open reading frame unlikely to encode a protein, based on available experimental and comparative sequence data; partially overlaps the verified gene TKL2 |
| YBL075C | SSA3 | 2.24 | 0.0001 | ATPase involved in protein folding and the response to stress; plays a role in SRP-dependent cotranslational protein-membrane targeting and translocation; member of the heat shock protein 70 (HSP70) family; localized to the cytoplasm |
| YCR021C | HSP30 | 2.26 | 1.95e-06 | Hydrophobic plasma membrane localized, stress-responsive protein that negatively regulates the H(+)-ATPase Pma1p; induced by heat shock, ethanol treatment, weak organic acid, glucose limitation, and entry into stationary phase |
| YBR117C | TKL2 | 2.28 | 9.56e-08 | Transketolase, similar to Tkl1p; catalyzes conversion of xylulose-5-phosphate and ribose-5-phosphate to sedoheptulose-7-phosphate and glyceraldehyde-3-phosphate in the pentose phosphate pathway; needed for synthesis of aromatic amino acids |
| YDR034W-B | . | 2.30 | 1.04e-07 | Protein of unknown function; green fluorescent protein (GFP)-fusion protein localizes to the cell periphery |
| YER103W | SSA4 | 2.31 | 2.20e-08 | Heat shock protein that is highly induced upon stress; plays a role in SRP-dependent cotranslational protein-membrane targeting and translocation; member of the HSP70 family; cytoplasmic protein that concentrates in nuclei upon starvation |
| YJR004C | SAG1 | 2.37 | 3.85e-08 | Alpha-agglutinin of alpha-cells, binds to Aga1p during agglutination, N-terminal half is homologous to the immunoglobulin superfamily and contains binding site for a-agglutinin, C-terminal half is highly glycosylated and contains GPI anchor |
| YHR139C | SPS100 | 2.50 | 3.98e-08 | Protein required for spore wall maturation; expressed during sporulation; may be a component of the spore wall |
| YCL025C | AGP1 | 2.54 | 4.90e-08 | Low-affinity amino acid permease with broad substrate range, involved in uptake of asparagine, glutamine, and other amino acids; expression is regulated by the SPS plasma membrane amino acid sensor system (Ssy1p-Ptr3p-Ssy5p) |
| YJL170C | ASG7 | 2.55 | 6.74e-07 | Protein that regulates signaling from a G protein beta subunit Ste4p and its relocalization within the cell; specific to a-cells and induced by alpha-factor |
| YBR040W | FIG1 | 2.55 | 1.58e-09 | Integral membrane protein required for efficient mating; may participate in or regulate the low affinity Ca2+ influx system, which affects intracellular signaling and cell-cell fusion during mating |
| YMR169C | ALD3 | 2.65 | 2.55e-07 | Cytoplasmic aldehyde dehydrogenase, involved in beta-alanine synthesis; uses NAD+ as the preferred coenzyme; very similar to Ald2p; expression is induced by stress and repressed by glucose |
| YHR033W | . | 2.65 | 2.82e-08 | Putative protein of unknown function; epitope-tagged protein localizes to the cytoplasm |
| YDL218W | . | 2.77 | 3.68e-08 | Putative protein of unknown function; YDL218W transcription is regulated by Azf1p and induced by starvation and aerobic conditions |
| YMR304C-A | . | 2.82 | 1.24e-10 | Dubious open reading frame unlikely to encode a protein, based on available experimental and comparative sequence data; partially overlaps the verified gene SCW10 |
| YIL015W | BAR1 | 2.86 | 1.06e-08 | Aspartyl protease secreted into the periplasmic space of mating type a cells, helps cells find mating partners, cleaves and inactivates alpha factor allowing cells to recover from alpha-factor-induced cell cycle arrest |
| YDL223C | HBT1 | 2.98 | 2.97e-09 | Substrate of the Hub1p ubiquitin-like protein that localizes to the shmoo tip (mating projection); mutants are defective for mating projection formation, thereby implicating Hbt1p in polarized cell morphogenesis |
| YPL223C | GRE1 | 3.03 | 4.54e-06 | Hydrophilin of unknown function; stress induced (osmotic, ionic, oxidative, heat shock and heavy metals); regulated by the HOG pathway |
| YGL032C | AGA2 | 3.40 | 2.59e-10 | Adhesion subunit of a-agglutinin of a-cells, C-terminal sequence acts as a ligand for alpha-agglutinin (Sag1p) during agglutination, modified with O-linked oligomannosyl chains, linked to anchorage subunit Aga1p via two disulfide bonds |
| YKR013W | PRY2 | 3.53 | 9.11e-09 | Protein of unknown function, has similarity to Pry1p and Pry3p and to the plant PR-1 class of pathogen related proteins |
| YMR305C | SCW10 | 3.62 | 8.50e-07 | Cell wall protein with similarity to glucanases; may play a role in conjugation during mating based on mutant phenotype and its regulation by Ste12p |
| YNR044W | AGA1 | 3.75 | 8.08e-11 | Anchorage subunit of a-agglutinin of a-cells, highly O-glycosylated protein with N-terminal secretion signal and C-terminal signal for addition of GPI anchor to cell wall, linked to adhesion subunit Aga2p via two disulfide bonds |
| YFL014W | HSP12 | 3.90 | 6.49e-11 | Plasma membrane localized protein that protects membranes from desiccation; induced by heat shock, oxidative stress, osmostress, stationary phase entry, glucose depletion, oleate and alcohol; regulated by the HOG and Ras-Pka pathways |
| YHR096C | HXT5 | 4.09 | 4.59e-09 | Hexose transporter with moderate affinity for glucose, induced in the presence of non-fermentable carbon sources, induced by a decrease in growth rate, contains an extended N-terminal domain relative to other HXTs |
